# Identification of EMT-Related Genes and Prognostic Signature With Significant Implications on Biological Properties and Oncology Treatment of Lower Grade Gliomas

**DOI:** 10.3389/fcell.2022.887693

**Published:** 2022-05-17

**Authors:** Jiasheng Wu, Jinru He, Jiheng Zhang, Hang Ji, Nan Wang, Shuai Ma, Xiuwei Yan, Xin Gao, Jianyang Du, Zhihui Liu, Shaoshan Hu

**Affiliations:** ^1^ Department of Neurosurgery, Emergency Medicine Center, Zhejiang Provincial People’s Hospital, Affiliated to Hangzhou Medical College, Hangzhou, China; ^2^ Department of Neurosurgery, The Second Affiliated Hospital of Harbin Medical University, Harbin, China; ^3^ School of Life Science and Technology, Harbin Institute of Technology, Harbin, China; ^4^ Department of Neurosurgery, Shandong Provincial Hospital Affiliated to Shandong First Medical University, Jinan, China; ^5^ Translational Medicine Research and Cooperation Center of Northern China, Heilongjiang Academy of Medical Sciences, Harbin, China

**Keywords:** epithelial-mesenchymal transition, lower grade gliomas, immune, stemness, adjacent non-tumor tissues, oncology treatment

## Abstract

The epithelial-mesenchymal transition (EMT) is an important process that drives progression, metastasis, and oncology treatment resistance in cancers. Also, the adjacent non-tumor tissue may affect the biological properties of cancers and have potential prognostic implications. Our study aimed to identify EMT-related genes in LGG samples, explore their impact on the biological properties of lower grade gliomas (LGG) through the multi-omics analysis, and reveal the potential mechanism by which adjacent non-tumor tissue participated in the malignant progression of LGG. Based on the 121 differentially expressed EMT-related genes between normal samples from the GTEx database and LGG samples in the TCGA cohort, we identified two subtypes and constructed EMTsig. Because of the genetic, epigenetic, and transcriptomic heterogeneity, malignant features including clinical traits, molecular traits, metabolism, anti-tumor immunity, and stemness features were different between samples with C1 and C2. In addition, EMTsig could also quantify the EMT levels, variation in prognosis, and oncology treatment sensitivity of LGG patients. Therefore, EMTsig could assist us in developing objective diagnostic tools and in optimizing therapeutic strategies for LGG patients. Notably, with the GSVA, we found that adjacent non-tumor tissue might participate in the progression, metastasis, and formation of the tumor microenvironment in LGG. Therefore, the potential prognostic implications of adjacent non-tumor tissue should be considered when performing clinical interventions for LGG patients. Overall, our study investigated and validated the effects of EMT-related genes on the biological properties from multiple perspectives, and provided new insights into the function of adjacent non-tumor tissue in the malignant progression of LGG.

## Graphical Abstract

The mechanism of how EMT-related genes affected the biological properties of LGG and the predictive capability of EMTsig.
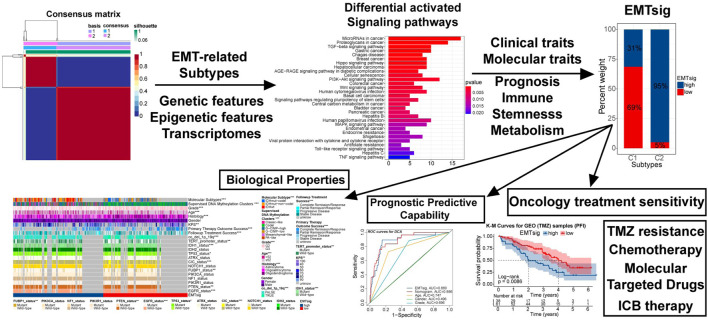

## Highlights

• The 1184 EMT-related genes summarized in the public databases were used to identify the EMT-related genes in LGG samples.
• Based on 121 differentially expressed EMT-related genes, we identified two distinct expression patterns of these genes and clustered LGG samples into two subtypes.
• Biological properties like immune and stemness features, clinical and molecular traits, metabolism, and prognosis were significantly differed between the subtypes. These variations could be attributed to alterations in genetic, epigenetic, and transcriptomic features.
• Based on EMTsig and GSVA techniques, we revealed the potential functions that adjacent non-tumor tissue might potentially perform during the process of EMT formation and the malignant progression of LGG.
• EMTsig was suggestive for the sensitivity of chemotherapy (including TMZ), ICB therapy, and molecular targeted drug therapy, extending the clinical application prospects of EMTsig.

## 1 Introduction

The central nervous system (CNS) controls most of the physical and basic life activities. About 80% of the malignant tumors in the CNS are gliomas, which are characterized by easy metastasis, aggressiveness, high mortality, and are hard to be treated (Ostrom et al., 2014). In this study, we focused on WHO grade II/III gliomas and designated them by lower grade gliomas (LGG) (Suzuki et al., 2015). Compared with glioblastoma (GBM), LGG has a relatively better prognosis, accounting for approximately one-third of CNS malignancies (Ostrom et al., 2020). With the improved understanding of LGG, it was not mere tumor tissue but a chronic central nervous system disease with progressive and aggressive properties. LGG can migrate along with the white matter and eventually inevitably progress to GBM with a higher degree of malignancy (Buckner et al., 2017). Therefore, an in-depth understanding of the mechanisms of tumorigenesis and progression of LGG can help us to more comprehensively understand the heterogeneity of biological properties between patients and thus improve their clinical outcomes through individualized treatment.

The essence of epithelial-mesenchymal transition (EMT) is the developmental process by which resting cells acquire the ability to migrate (Greenburg and Hay, 1982). In the context of cancer, the EMT process is often reactivated, which leads to a rise in the invasive and metastatic capacity of cancer cells (Rhim et al., 2012; Krebs et al., 2017). EMT has a profound impact on the biological properties of cancers. For example, EMT is associated with the acquisition of stem cell characteristics of breast cancer cells (Mani et al., 2008). Also, EMT-related cancer stem cell transformation is often accompanied by resistance to oncology treatment (Zhou et al., 2017; Singh et al., 2018). In addition, EMT transcription factors (EMT-TFs) can induce resistance to chemotherapy or radiotherapy through various mechanisms such as resistance to apoptosis and altered drug metabolism (van Staalduinen et al., 2018). Therefore, the impact of EMT must be considered when developing individualized treatment plans. Similarly, EMT is involved in the formation of the immunosuppressive microenvironment, resulting in impaired anti-tumor immunity. For example, EMT can lead to disruption of immune synapses, eventually leading to impaired T cells CD8-mediated cellular immunity (Akalay et al., 2013). Moreover, EMT can upregulate the expression of immune checkpoints, which is related to immune escape and the sensitivity of immunotherapy (Chen et al., 2014; Lou et al., 2016; Ramesh et al., 2020). Thus, EMT appears to be involved in all aspects of cancer progression and is a huge challenge for oncology treatment. Notably, adjacent non-tumor tissue may play a specific role in cancer progression, EMT processes, and regulation of anti-tumor immunity (Jin et al., 2013; Aran et al., 2017; Wu et al., 2019; Yang et al., 2020; Gong et al., 2021). Therefore, it is necessary to explore the potential function of adjacent non-tumor tissue in the malignant progression of LGG. This can bring novel therapeutic opportunities for LGG patients in clinical applications. In addition, previous studies have attempted to reveal the prognostic impact of EMT on glioma. For example, Tao et al. constructed an EMT-related lncRNAs prognostic model based on glioma samples and explored the predictive capability of EMT-related lncRNAs for the immune features (Tao et al., 2021). However, this study has not fully explored the correlation of their prognostic model with genetic features, epigenetic modifications of DNA and RNA, stemness features, metabolism, the efficiency of radiotherapy and TMZ chemotherapy, and sensitivity to immune checkpoint blockade (ICB) therapy in LGG. To fill these gaps and identify a novel multifunctional EMT-related biomarker, we designed this study.

In this study, we identified 121 differentially expressed EMT-related genes between normal samples from the GTEx database and LGG samples in the TCGA cohort. Next, LGG samples in the TCGA cohort were clustered into two subtypes. We found malignant features including EMT levels, prognosis, clinical traits, molecular traits, metabolism, anti-tumor immunity, and stemness features significantly differed between samples with C1 and C2. Next, through multi-omics and functional enrichment analysis, we investigated the correlations of variations in genomes, transcriptomes, molecular functions, and cancer signaling pathways with EMT-related genes and elucidated the underlying genetic and epigenetic mechanisms. Furthermore, to quantify the individual variations, EMTsig was constructed. The activity of cancer-related molecular functions and signaling pathways varied across high and low EMTsig subgroups in both normal samples and LGG samples in the TCGA cohort. This not only pointed out that adjacent non-tumor tissue might participate in the malignant progression of LGG but also indicated that EMTsig was suggestive of the evolving trend of malignant features. In addition, EMTsig could predict the sensitivity of multiple chemotherapeutic agents, molecular targeted drugs, and immune checkpoint blockade (ICB) therapy. In summary, our study identified a new prognostic model based on EMT-related genes with comprehensive and accurate instructions on the biological features, clinical outcomes, and selection of individualized therapeutic strategies for LGG patients.

## 2 Materials and Methods

### 2.1 Preparation of Data

#### 2.1.1 Download Data

The multi-omics data involved in our study were obtained from public databases or Supplementary Material from the published studies. From the TCGA database, we downloaded RNA-seq matrices, copy number variation (CNV) information, clinical data, and somatic mutation profiles (Varscan) for all LGG samples (https://portal.gdc.cancer.gov/). The somatic mutation information of TCGA samples was obtained from the UCSC Xena database (http://xena.ucsc.edu/) (Goldman et al., 2020). The supervised DNA methylation clusters, molecular subtypes, and immunophenotype profiles of TCGA samples were obtained from the Supplementary Material of relevant studies (Brat et al., 2015; Ceccarelli et al., 2016; Malta et al., 2018). From the CGGA database, we selected three cohorts: mRNA-array_301, mRNAseq_325, and mRNAseq_693, and downloaded the relevant RNA-seq matrices and clinical data (http://www.cgga.org.cn/). RNA-seq matrices for normal cortical samples were obtained from the GTEx database (https://gtexportal.org/) (Consortium, 2013). From the GEO database, we obtained a cohort (GSE107850) containing LGG samples treated with TMZ (https://www.ncbi.nlm.nih.gov/geo/). This dataset provides RNA-seq matrices and progression-free interval (PFI) information for GEO samples. The gene sets used for the gene set variation analysis (GSVA) were downloaded from the GSEA database (http://www.gsea-msigdb.org/gsea/index.jsp).

#### 2.1.2 Preprocessing of Data

Firstly, we consolidated data from the same sources. A total of 509 TCGA samples simultaneously have clinical data, gene expression, and mutational information. As for the CGGA cohort, a total of 592 LGG samples had both gene expression and clinical information. In the GEO cohort, a total of 99 samples received TMZ treatment. In addition, 207 normal cortical samples were screened from the GTEx samples. The “normalizeBetweenArrays” function in the “limma” package was used to normalize the RNA-seq matrix before performing multi-database analysis so that subsequent results would be comparable.

### 2.2 Identification of Subtypes With EMT-Related Genes

The dbEMT 2.0 database contains 1,184 EMT-related genes (http://www.dbemt.bioinfo-minzhao.org/) (Zhao et al., 2019). We used the thresholds of |logFC| > 1and FDR < 0.05 and screened for differential expressed EMT-related genes by using the “edgeR” package (Robinson et al., 2010). In the TCGA cohort, after removing genes that were completely unexpressed in the LGG samples, a total of 121 EMT-related genes were differentially expressed between normal and LGG samples. Non-negative matrix factorization (NMF) can compress huge data by decomposing a large non-negative matrix into two small non-negative matrices representing the number of data and data characteristics, which is an effective strategy for dimensionality reduction. Since the gene expression profiles are essentially non-negative matrices, the NMF method is widely used for the identification of molecular patterns for high-dimensional genomic data. In this paper, this part is performed with the “NMF” package. Based on 121 differentially expressed EMT-related genes, two reliable subtypes were clustered in the TCGA cohort, which were named C1 and C2.

### 2.3 ssGSEA, CIBERSORT, and ESTIMATE

The ssGSEA allows the definition of enrichment scores that indicates the absolute enrichment of gene sets in each sample within the given data sets. Our study involves multiple gene sets which represent immune functions, metabolism, epithelial-mesenchymal transition (EMT), extracellular matrix (ECM), and cancer signaling pathways. These gene sets were obtained from public databases or Supplementary Material from published studies (http://matrisome.org/; https://www.immport.org/; https://www.rndsystems.com/) (Naba et al., 2016; Bhattacharya et al., 2018; Mariathasan et al., 2018). With the “GSVA” package, we calculated the ssGSEA score levels for these gene sets.

Essentially, both the CIBERSORT and ESTIMATE algorithms are extensions of ssGSEA. The CIBERSORT algorithm was used to forecast the infiltration of 22 kinds of immune cells, and the main program and gene sets of immune cells were obtained from GitHub and the Supplementary Material of the related study (https://github.com/) (Newman et al., 2015). ESTIMATE algorithm is based on the “estimate” package for evaluating the tumor purity (Yoshihara et al., 2013).

### 2.4 WGCNA

Weighted correlation network analysis (WGCNA) can be used to identify gene sets with highly synergistic changes, and the “WGCNA” package is required for its implementation (Langfelder and Horvath, 2008). The principle of WGCNA is to compress multiple gene reactions into a minimum number of gene modules using the unsupervised clustering method. Based on the assumption that highly correlated genes within modules are involved in common biological processes, Eigengenes (the first principal component within modules) are used to quantify the correlation between selected indices and modules. In this study, we aimed to identify differentially expressed immune and stemness-related genes between the C1 and C2 subtypes. Similarly, with the “edgeR” package, we identified 1,912 differentially expressed genes (DEGs) between C1 and C2 samples (|logFC| > 1, FDR < 0.05). We chose the ESTIMATE score, mDNAsi, and mRNAsi as the immune stemness indices, and constructed the weighted network in combination with DEGs. Next, a scale-free topology system was constructed by using the optimal R2 = 0.88 and a soft threshold (*β* = 4). Based on the “TOMSimilarity” function, the proximity matrix was converted into a topology overlap matrix. Next, by hierarchical clustering, we calculated the module Eigengenes (MEs). By combining modules with overall gene counts <30 and MEs correlation coefficients > 0.75, a total of nine modules were finally retained. MEbrown, MEgreen, and MEturquoise modules were the main modules of the mDNAsi, mRNAsi, and ESTIMATE score respectively. We screened for Eigengenes in the three main modules. Genes meeting the threshold (Gene Significance (GS) > 0.5 and Module Membership (MM) > 0.7) were retained as Eigengenes of the corresponding modules.

### 2.5 Functional Enrichment Analysis

Before the analysis, the “org.Hs.eg.db” package was used to convert the gene symbols into ensemble IDs. Gene Ontology (GO) and the Kyoto Protocol Encyclopedia of Genes and Genomes (KEGG) functional enrichment analyses were performed with the “clusterProfiler” package (Yu et al., 2012). Only GO and KEGG pathways with *p* < 0.05 were retained. GSVA is performed based on the “GSVA” package, and the input files include the RNA-seq matrix and the relevant gene set files for the GO and KEGG pathways downloaded from GSEA (https://www.gsea-msigdb.org/gsea/).

### 2.6 Genetic Variation Analysis

For the analysis of CNV, we used the “limma” package for the variance analysis of CNV types and frequencies of EMT-related genes between samples with C1 and C2. The interaction relationships between genes were obtained from the String database (https://string-db.org/). The “RCircos” package was used to plot the RCircos plot containing information about gene positions, CNV frequencies, gene expression, and interactions between EMT-related genes. In addition, some specific CNV events, such as Chr7 gain, Chr10 loss, Chr19/20 co-gain, EGFR amplification, PDGFRA amplification, and CDKN2A/B homozygous deletion have a significant impact on the prognosis of gliomas. Therefore, in the TCGA cohort, we verified whether these events were differentially distributed between the C1 and C2 subtypes. Information on Chr7 gain, Chr10 loss, and Chr19/20 co-gain was obtained from published studies (Ceccarelli et al., 2016). information on EGFR amplification, PDGFRA amplification, and CDKN2A/B homozygous deletion was obtained from the CNV information downloaded from the TCGA database.

As for the analysis of somatic mutation information, we used the “maftools” package in this part (Mayakonda et al., 2018). Firstly, the information about deletion (DEL), insertion (INS), single-nucleotide variants (SNV), single-nucleotide polymorphism (SNP), somatic mutation counts, and variant allele fraction (VAF) was extracted from the somatic mutation profile. The Mann-Whitney *U* test was used to analyze the variation of these indices between the C1 and C2 subtypes. Waterfall plots were plotted by using the “oncoplot” function. The lollipop plots were painted with the “lollipopPlot” function. Mutation patterns between the C1 and C2 subtypes were compared with Fisher’s exact test, and the differences in the 15 most frequently mutated genes were plotted by using the “forestplot” function. Correlations of 30 top mutated genes were obtained by Fisher’s exact test by using the CoMEt algorithm provided by the “somaticInteractions” function. Identification of driver genes was performed by the “oncodrive” function. The enrichment levels and activities of cancer signaling pathways were visualized by the “OncogenicPathways” function.

### 2.7 Driver Genes for DNA Methylation

We downloaded the DNA methylation profiles of TCGA samples from the UCSC Xena database (https://xena.ucsc.edu/). The “MethylMix” package was used to identify the driver genes for DNA methylation (Gevaert, 2015). The threshold for the driver genes was |cor| > 0.3 and FDR < 0.05. A total of 617 driver genes were identified.

### 2.8 Construction of EMTsig

Based on 121 differentially expressed EMT-related genes, overall survival (OS) time, and OS status, EMTsig was constructed with the least absolute shrinkage and selection operator (LASSO) regression. Firstly, we equally divided the TCGA samples into the train and test sets. After examination, there was no difference in clinical traits between the training and test sets. Next, based on the train set, the 10-fold cross-validation was used to determine the best penalty coefficient (log(λ) = −2.7). With this λ value, only four genes had nonzero coefficients. Therefore, these genes were identified as the characteristic variables (principal components) of 121 differentially expressed EMT-related genes. After being validated by univariate and multivariate Cox analysis, all these four genes retained prognostic significance. Therefore, EMTsig was constructed according to the following equation: $EMTsig=\sum_{i=1}^{n}Coef\left(gene_{i}\right)*\exp\left(gene_{i}\right)$ . Notably, the “Coef” represented the coefficients corresponding to these four genes at log(λ) = −2.7, and the “exp” represented the expression of these four genes. As for the CGGA cohort, the GEO cohort, and normal samples, we calculated the EMTsig for each sample with the identical formula.

### 2.9 Sensitivity of Oncology Treatment

The relationship of chemotherapeutic agents and molecular targeted drugs with gene expression was obtained from the CellMiner database (https://discover.nci.nih.gov/cellminer/home.do) (Reinhold et al., 2012). In our study, we selected only drugs with *p* < 0.05 and FDA approval. In addition, to more accurately determine the correlation between EMTsig and the sensitivity of ICB therapy, we used two analytical tools from the websites. The ImmuCellAI algorithm was used to predict the infiltration levels of 24 types of lymphocytes in tumor tissue (http://bioinfo.life.hu-st.edu.cn/web/ImmuCellAI/) (Miao et al., 2020). The TIDE algorithm can predict the degree of impaired anti-tumor immunity and the infiltration levels of several immunosuppressive cells (http://tide.dfci.harvard.edu./) (Jiang et al., 2018). Combined with the relationship of EMTsig with TMB, infiltration levels of lymphocytes, and the expression of immune checkpoints, the effect of EMTsig on the sensitivity of ICB therapy can be accurately determined.

### 2.10 Software and Statistical Methods

Since only two subtypes were identified in LGG samples in our study. Therefore, the Mann-Whitney *U* test was used to compare differences between subtypes or subgroups. Correlations between variables were verified with the Spearman correlation analysis. The survival analysis was based on the Kaplan-Meier (K-M) method and the log-rank test. The Cox analysis was used to elucidate the correlation between variables and the prognosis of patients. The univariate Cox analysis was used to detect whether the variables were risk factors for prognosis, while the multivariate Cox analysis was used to determine whether the variables could be used as independent prognostic biomarkers. The accuracy of prognostic biomarkers was determined with ROC curves and the area under the curves (AUC). The net benefit on survival of LGG patients between different prognostic biomarkers was determined with decision curve analysis (DCA).

This study was conducted based on R version 4.1.1. The “pheatmap” package was used for plotting heatmaps. “ggplot2”, “ggpubr”, “ggExtra”, “plyr”, and “reshape2” packages could be used for plotting multiple figures, such as box plots, bar plots, and scatter diagrams. Forest plots were painted with the “forestplot” package, and ROC curves were plotted by the “timeROC” program. Principal component analysis (PCA) was implemented with the “limma” package and could be visualized with the “ggplot2” package. The prognostic network for selected writers of post-transcriptional RNA modification patterns were created with the “igraph” and “psych” packages. The upset plot was generated with the “upsetR” package. The nomogram was developed based on the “regplot” package. The “rms” package was required for the generation of calibration curves. K-M curves were plotted by the “survival” and “survminer” packages. The DCA and creation of decision curves were based on the “ggDCA” package. In addition, Perl scripts participated in the pre-processing of data (Strawberry-Perl-5.32.1.1).

## 3 Results

### 3.1 Identification of Subtypes Based on EMT-Related Genes

**FIGURE 1:**
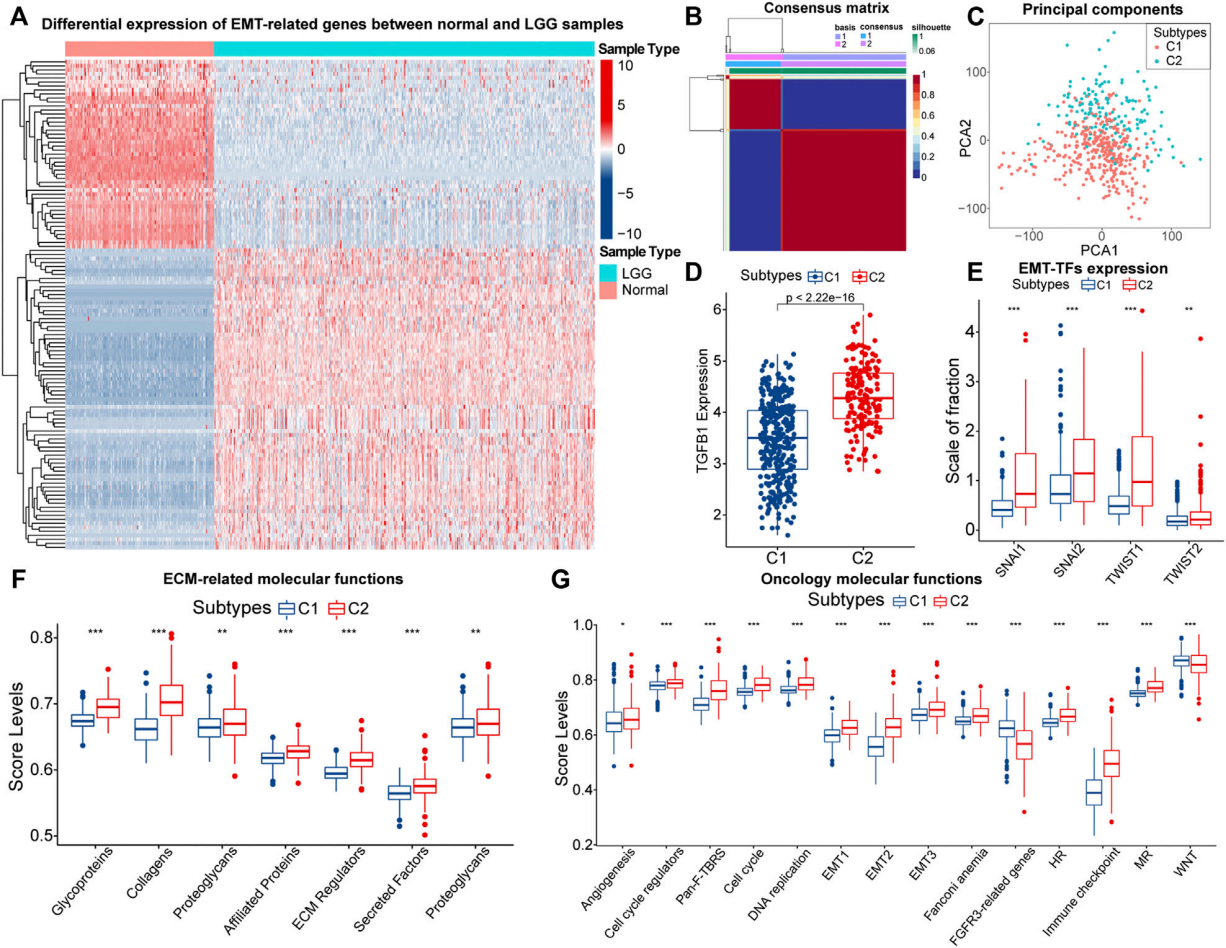
The heatmap of differential expressed EMT-related genes between normal and LGG samples **(A)**. Consensus matrix of NMF clusters **(B)**. Differences in principal components between the C1 and C2 subtypes **(C)**. Differences in TGF-β (TGFB1) **(D)**, SNAI1/2 and TWIST1/2 **(E)**, expression between samples with C1 and C2. Differences in ssGSEA score levels of gene sets related to the remodeling of extracellular matrix between samples with C1 and C2 **(F)**. Differences in ssGSEA score levels of gene sets related to oncology molecular functions between samples with C1 and C2 **(G)**. Notes for abbreviations: homologous recombination (HR); mismatch repair (MR). The identification of differentially expressed genes was based on the “edgeR” method. In the box plots, *p* < 0.05 was indicated by “*”, *p* < 0.01 was indicated by “**”, *p* < 0.001 was indicated by “***”, and the statistical analysis was performed by the Mann-Whitney *U* test.

A total of 1,184 EMT-related genes were used in this study. By removing genes that were completely unexpressed in LGG samples and using |logFC| > 1and FDR < 0.05 as the threshold, 121 differentially expressed EMT-related genes between normal samples from the GTEx database and LGG samples in the TCGA cohort were identified (Figure 1A; Supplementary Table 1). NMF rank survey suggested that the optimal rank value was 2 (Supplementary Figure S2A). According to the rank value, LGG samples in the TCGA cohort were clustered into two subtypes which were labeled as C1 and C2 (Figure 1B; Supplementary Table 2). As expected, the principal component significantly differed between samples with C1 and C2 (Figure 1C). Thus, LGG samples could be reliably clustered into two subtypes.

Next, we explored the differences in EMT levels between samples with C1 and C2. Compared to C1, the expression of TGF-β (TGFB1) was significantly higher in samples with C2 (Figure 1D). The EMT process is mediated by EMT-TFs such as Snail (SNAI1), slug (SNAI2), and TWIST1/2, and they were equally highly expressed in samples with C2 (Figure 1E). In addition, EMT is often accompanied by the remodeling of the extracellular matrix (ECM). Among the matrix metalloproteinases (MMPs), like MMP2/3/9 are closely related to the decomposition of the basement membrane and remodeling of the ECM (Miyoshi et al., 2004). These three genes were highly expressed in samples with C2 (Supplementary Figure S2B). Also, Figure 1F indicated that the ECM-related molecular functions were significantly hyper-activated in samples with C2. Notably, the rise in cell motility is often accompanied by alterations in adhesion molecules and the cytoskeleton. In samples with C2, the expression of E-cadherin (CDH1) was downgraded, and N-cadherin (CDH2) was significantly highly expressed (Supplementary Figure S2C). Moreover, vimentin (VIM) mainly maintains cytoskeletal integrity and is important for cell motility and metastatic spread (Costigliola et al., 2017). Similarly, VIM was highly expressed in samples with C2 (Supplementary Figure S2D). Finally, we found that score levels of EMT-related gene sets, such as Pan-F-TRBS, angiogenesis, and EMT1/2/3 were higher in samples with C2 (Figure 1G). However, the score levels corresponding to WNT signaling pathways and FGFR3-related gene sets were slightly lower in samples with C2, which might be attributed to the activation of the potential negative feedback regulatory mechanism against the EMT process. In summary, among these two EMT-related subtypes, multiple pieces of evidence suggested that samples with C2 had higher EMT levels.

### 3.2 The Relation of Subtypes With the Prognosis and Malignant Features of LGG

**FIGURE 2:**
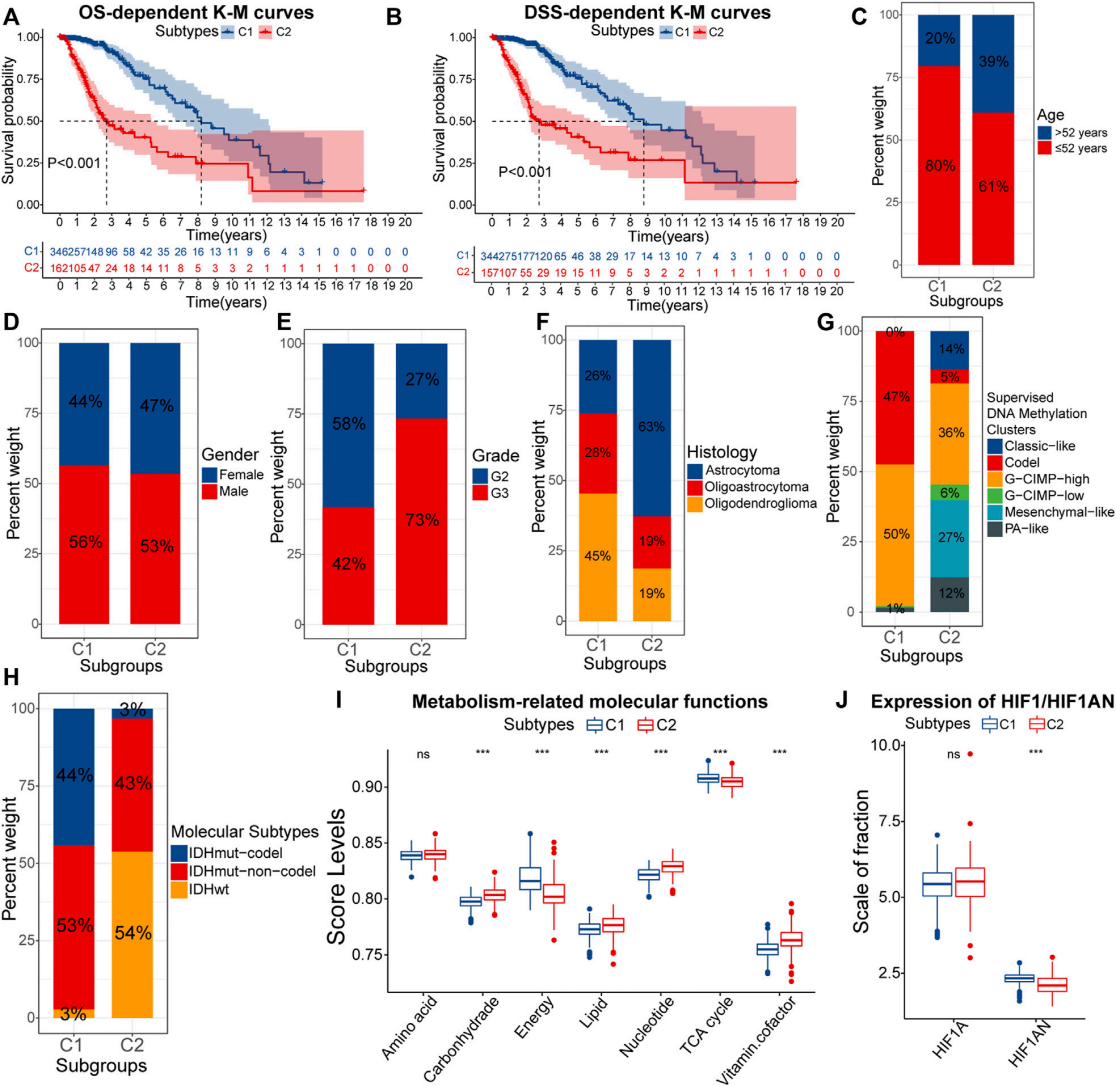
K-M curves for the overall survival (OS) **(A)** and disease-specific survival (DSS) **(B)** between samples with C1 and C2. Differences in the composition ratios of age **(C)**, gender **(D)**, grade **(E)**, histology **(F)**, supervised DNA methylation clusters **(G)**, and molecular subtypes **(H)** between samples with C1 and C2. Differences in ssGSEA score levels of gene sets related to the metabolism between samples with C1 and C2 **(I)**. Differences in the expression of HIF1A and HIF1AN between samples with C1 and C2 **(J)**. In the box plots, *p* < 0.05 was indicated by “*”, *p* < 0.01 was indicated by “**”, *p* < 0.001 was indicated by “***”, and the statistical analysis was performed by the Mann-Whitney *U* test.

Further, we explored and validated the prognostic implications of EMT-related genes. The K-M curves showed that the median OS of samples with C2 was less than 3years, much lower than 8.2 years in samples with C1 (Figure 2A). After eliminating the non-disease mortality, samples with C2 still had a worse prognosis (Figure 2B). Therefore, obtained the C2 subtype always indicated adverse clinical outcomes in LGG patients. To further investigate the mechanism behind it, we next verified the differential distribution of malignant features of LGG, including clinical traits, molecular traits, and metabolic status between EMT-related subtypes.

As for clinical traits, there was no difference in susceptibility to EMT between genders, but samples of senior (>52 years) and high grade (G3) were more likely to acquire the C2 subtype (Figures 2C–E). Next, we evaluated the correlations between molecular traits between molecular traits and EMT-related subtypes. Astrocytomas occupied 63% of the C2 samples, much higher than 26% in C1 (Figure 2F). Epidemiological evidence suggests that astrocytomas have a lower 5-year survival rate compared with oligoastrocytomas and oligodendrogliomas (Ostrom et al., 2014). Next, among the six supervised DNA methylation clusters, a total of 97% samples with C1 had G-CIMP-high and Codel, while Mesenchymal-like was almost exclusively present in samples with C2 (Figure 2G). Compared to the other four subtypes, samples with G-CIMP-high and Codel have the best prognosis (Ceccarelli et al., 2016). Also, the high proportion of Mesenchymal-like subtypes further confirmed that C2 samples had higher EMT levels. As for the three molecular subtypes, IDHwt is a risk factor for the prognosis, but IDHmut-codel is a protective factor (Brat et al., 2015). IDHwt and IDHmut-codel accounted for 54% and 3% of samples with C2, compared to 3% and 44% of samples with C1 (Figure 2H). Thus, samples with C2 tended to have clinical and molecular traits with a worse prognosis.

Furthermore, the metabolic abnormality is one of the hallmarkers of cancers (Hanahan and Weinberg, 2011). Figure 2I presented the activity of metabolism-related molecular functions between samples with C1 and C2. Lipids are essential nutrients for glioma cells (Gimple et al., 2019). Thus, hyper-activated lipid metabolism in samples with C2 was more favorable for the progression of LGG. Similarly, In C2 samples, the carbohydrate metabolic activity did not match the activity of tricarboxylic acid (TCA) and energy metabolism. This might be attributed to the higher degree of hypoxia in samples with C2, which led to a further shift in the metabolic model of LGG cells toward aerobic glycolysis. In fact, hypoxia-inducible factor 1-alpha inhibitor (HIF1AN) was under-expressed in samples with C2 (Figure 2J). In addition, vitamin cofactors, such as NADPH may limit the proliferation of cancer cells (Vander Heiden et al., 2009). Thus, elevated activity of vitamin cofactor metabolism might indicate a more vigorous proliferation of cancer cells. In summary, samples with C2 owned more severe metabolic abnormality. The accumulation of multiple risk factors might be related to the poor prognosis of samples with C2.

### 3.3 The Variations in Immune and Stemness Features Between Samples With Different Subtypes

Previous studies have pointed out that EMT is potentially correlated with the remodeling of anti-tumor immunity and stemness features (Mani et al., 2008; Akalay et al., 2013; Chen et al., 2014; Lou et al., 2016). Also, the EMT-related genes may additionally affect other biological properties which can significantly affect the prognosis of LGG patients. Therefore, we further investigated the variations in immune and stemness characteristics between samples with C1 and C2.

**FIGURE 3:**
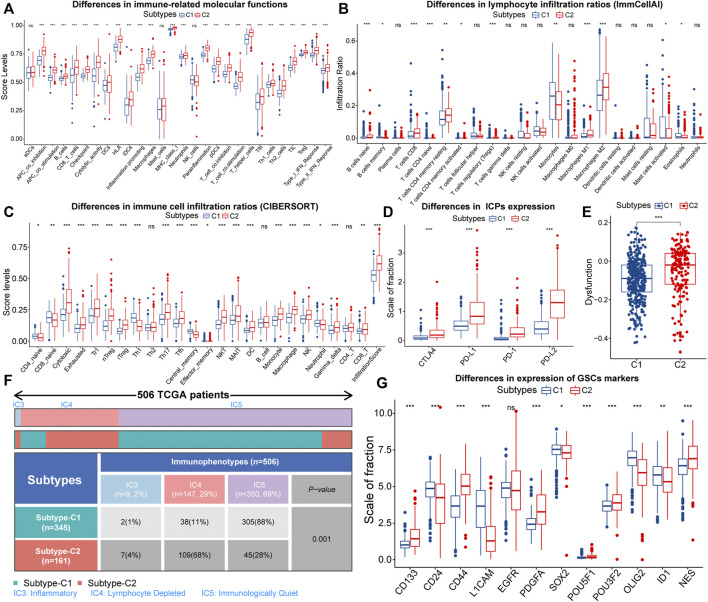
Differences in ssGSEA score levels of gene sets related to immune functions between samples with C1 and C2 **(A)**. Differences of infiltration ratios of 22 immune cells (CIBERSORT) **(B)** and 24 lymphocytes (ImmuCellAI) **(C)** between samples with C1 and C2. Differences in the expression of immune checkpoints **(D)** and dysfunction score **(E)** between samples with C1 and C2. Differential distribution of the immunophenotypes between samples with C1 and C2 **(F)**. Differences in the expression of 12 glioma stem cells (GSCs) markers between samples with C1 and C2 **(G)**. In the box plots, *p* < 0.05 was indicated by “*”, *p* < 0.01 was indicated by “**”, *p* < 0.001 was indicated by “***”, and the statistical analysis was performed by the Mann-Whitney *U* test.

Firstly, we characterized immune features between different subtypes. About immune-related signals, both pro-tumor and anti-tumor signals were upregulated in samples with C2 (Figure 3A). Therefore, EMT might have a two-sided effect on anti-tumor immunity. Next, we depicted the infiltration of 22 immune cells in the TCGA cohort. The correlation between most immune cells was not strong, and only a few pro-tumor cells displayed an intense inhibition of anti-tumor cell infiltration (Supplementary Figure S3A). This might indicate impaired anti-tumor immunity in LGG patients. Also, the proportion of pro-tumor cells, such as macrophages M2, Tregs, B cells naive, mast cells resting, and T cells CD4 memory resting was higher in samples with C2. However, T cells CD8, as one kind of anti-tumor cell, was highly infiltrated in samples with C2 (Figure 3B). Next, we further evaluated the infiltration patterns of lymphocytes with the ImmuCellAI algorithm (Figure 3C). We noted that the cytotoxic levels and infiltration levels cytotoxic-related cell like CD8 T cells, NKT, MAIT, Th17, and Tfh were higher in samples with C2. However, the proportion of cells that can strongly suppress the tumor immunity, such as nTreg and iTreg, increased in parallel in samples with C2. Notably, the dysfunction score and the expression of immune checkpoints were higher in samples with C2, signifying higher immune escape levels (Figures 3D,E). Therefore, the T-cell exhaustion levels in samples with C2 might be higher. In addition, the immune and stromal scores were significantly higher in samples with C2, implying that these samples with lower tumor purity (Supplementary Figures S3B,C). In terms of TCGA immunophenotypes, 68% of samples with C2 possessed Lymphocyte Depleted (IC4), whereas samples with C1 were predominantly Immunologically Quiet (IC5) (Figure 3F). Notably, samples with IC4 had the most confounding immune features and the worst prognosis (Thorsson et al., 2018). In summary, the anti-tumor immunity might be more severely impaired in samples with C2.

Next, we evaluated the stemness features of samples with C1 and C2. Referring to previous studies, mDNAsi and mRNAsi are a set of stemness indices obtained based on the OCLR machine learning algorithm, where mDNAsi reflects the epigenetic features of stem cells, and mRNAsi indicates the gene expression features of stem cells (Malta et al., 2018). Samples with high mDNAsi and low mRNAsi had a better prognosis in LGG (Supplementary Figures S3D,E). Compared to C1, samples with C2 had higher mDNAsi and lower mRNAsi (Supplementary Figures S3F,G). Notably, CD133 is an iconic marker of GSCs and tended to be highly expressed in the GBM with a higher degree of malignancy. (Singh et al., 2004; Du et al., 2020). It was highly expressed in samples with C2 (Figure 3G). Meanwhile, CD44 was highly expressed, while CD24 was lowly expressed in samples with C2 (Figure 3G). It is widely accepted that CD44^high^ and CD24^low^ cells are cancer stem cells (Mani et al., 2008). Thus, the C2 subtype might related to the accumulation of stemness properties by LGG cells, which could accelerate the malignant progression of LGG and lead to a poor prognosis.

### 3.4 Functional Annotation of Eigengenes Based on WGCNA

To investigate the specific genes and underlying biological functions that affected the immune and stemness features, we used weighted correlation network analysis (WGCNA) to construct co-expression networks and annotated the functions of Eigengenes. The ESTIMATE score is the sum of the immune score and the stromal score, and it varied between samples with C1 and C2 (Supplementary Figure S3H). Therefore, we selected the ESTIMATE score as the index to represent immune features. Similarly, referred to the relationship of mDNAsi and mRNAsi with stemness features, both of them were selected as the stemness indices.

**FIGURE 4:**
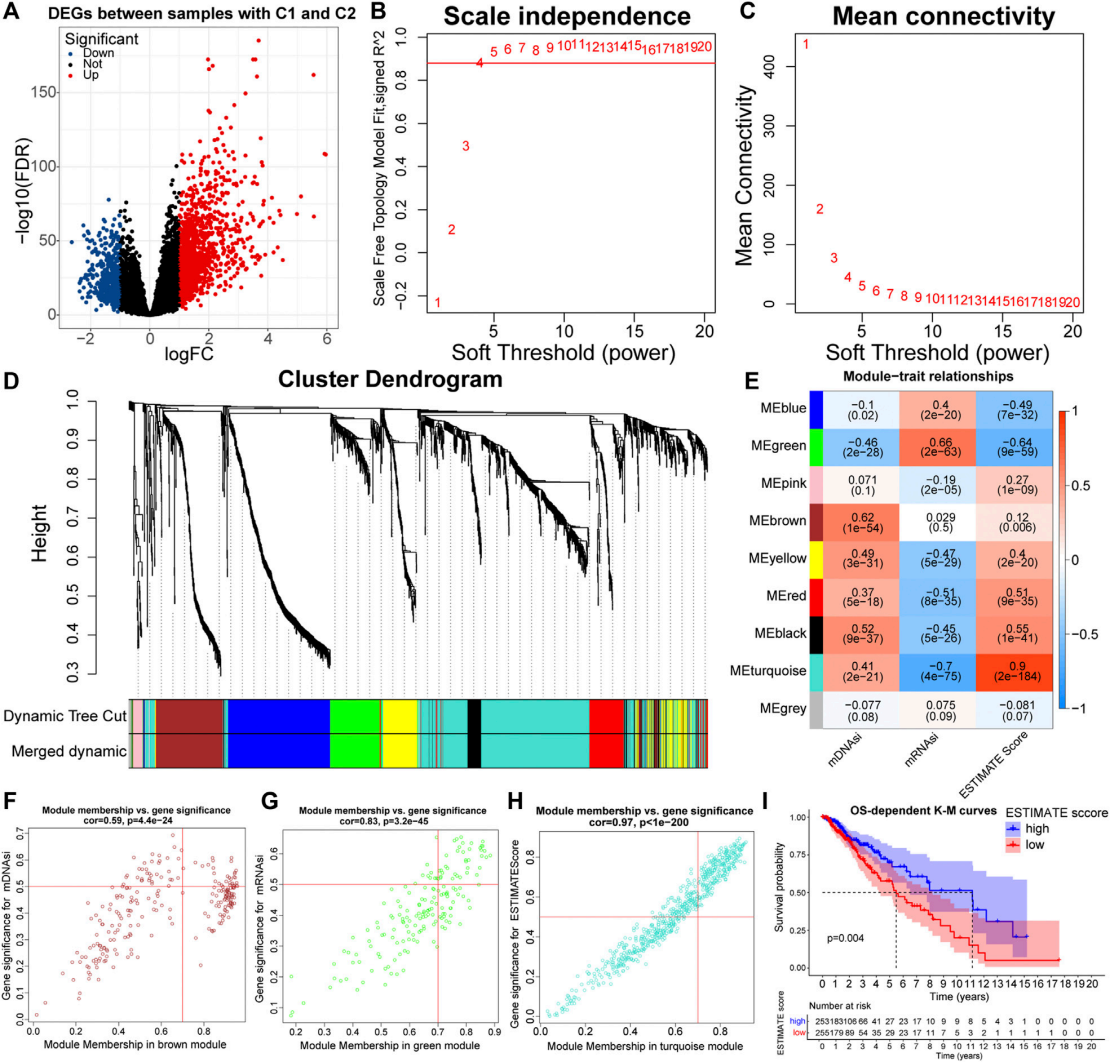
Volcano plot of differentially expressed genes (DEGs) between samples with C1 and C2 **(A)**. Relationship between the scale independence (*R* ^2^) **(B)** and mean connectivity **(C)** with the soft threshold (β). The branches of the cluster dendrogram correspond to the different gene modules. Each leaf on the cluster dendrogram corresponds to a gene, and the colored row represents a color-coded module that contains a group of highly connected genes **(D)**. Correlation coefficients of WGCNA gene modules with ESTIMATE score, mDNAsi, and mRNAsi **(E)**. Genes satisfying Gene Significance (GS) > 0.5 and Module Membership (MM) > 0.7 in the, MEbrown **(F)**, MEgreen **(G)**, and MEturquoise **(H)** modules were identified as Eigengenes. K-M curves between samples with high and low (clustering method: median values) ESTIMATE score **(I)**.

To keep the results more representative, we set FDR < 0.05 and |logFC| > 1 as the threshold, and a total of 1,912 differentially expressed genes (DEGs) were identified between samples with C1 and C2 (Figure 4A; Supplementary Table 3). These DEGs were used to build a weighted network. Next, a scale-free topology system was constructed by using the optimal *R* ^2^ = 0.88 and a soft threshold (*β* = 4) (Figures 4B,C). After merging modules with the disparity coefficient <0.25 and overall gene counts <30, nine modules were finally obtained (Figure 4D). By selecting modules with the highest correlation coefficients, the MEbrown, MEgreen, and MEturquoise modules were the main modules of mDNAsi, mRNAsi, and ESTIMATE score respectively (Figure 4E). Finally, Genes satisfying Gene Significance (GS) > 0.5 and Module Membership (MM) > 0.7 were identified as Eigengenes. A total of 31, 37, and 247 genes were identified as Eigengenes in the MEbrown, MEgreen, and MEturquoise modules (Figures 4F–H; Supplementary Table 4).

GO and KEGG functional enrichment analyses can reveal the potential biological functions of Eigengenes in main modules. Eigengenes in the MEturquoise module were closely related to the function of immune cells, immunoreactive substances, and recognition and presentation of antigens (Supplementary Figures S4A,B). Also, the prognosis samples with high ESTIMATE scores were relatively bad (Figure 4I). As for the MEbrown module, Eigengenes were highly enriched in the cell cycle and mitosis-related pathways, implying that mDNAsi might be associated with the proliferation of cancer cells (Supplementary Figures S4C,D). Eigengenes in the MEgreen module were highly enriched in GO pathways related to cell adhesion (Supplementary Figure S4E). These results revealed the mechanisms by which the immune and stemness features, prognosis, and malignancy differed between the C1 and C2 subtypes. Notably, the original study has demonstrated that mRNAsi is inversely correlated with EMT levels (Malta et al., 2018). Therefore, mDNAsi and mRNAsi might be a set of complementary stemness indices, and the conclusion of higher EMT levels in samples with C2 was further supported.

### 3.5 Genetic Variations Between Samples With Different Subtypes

**FIGURE 5:**
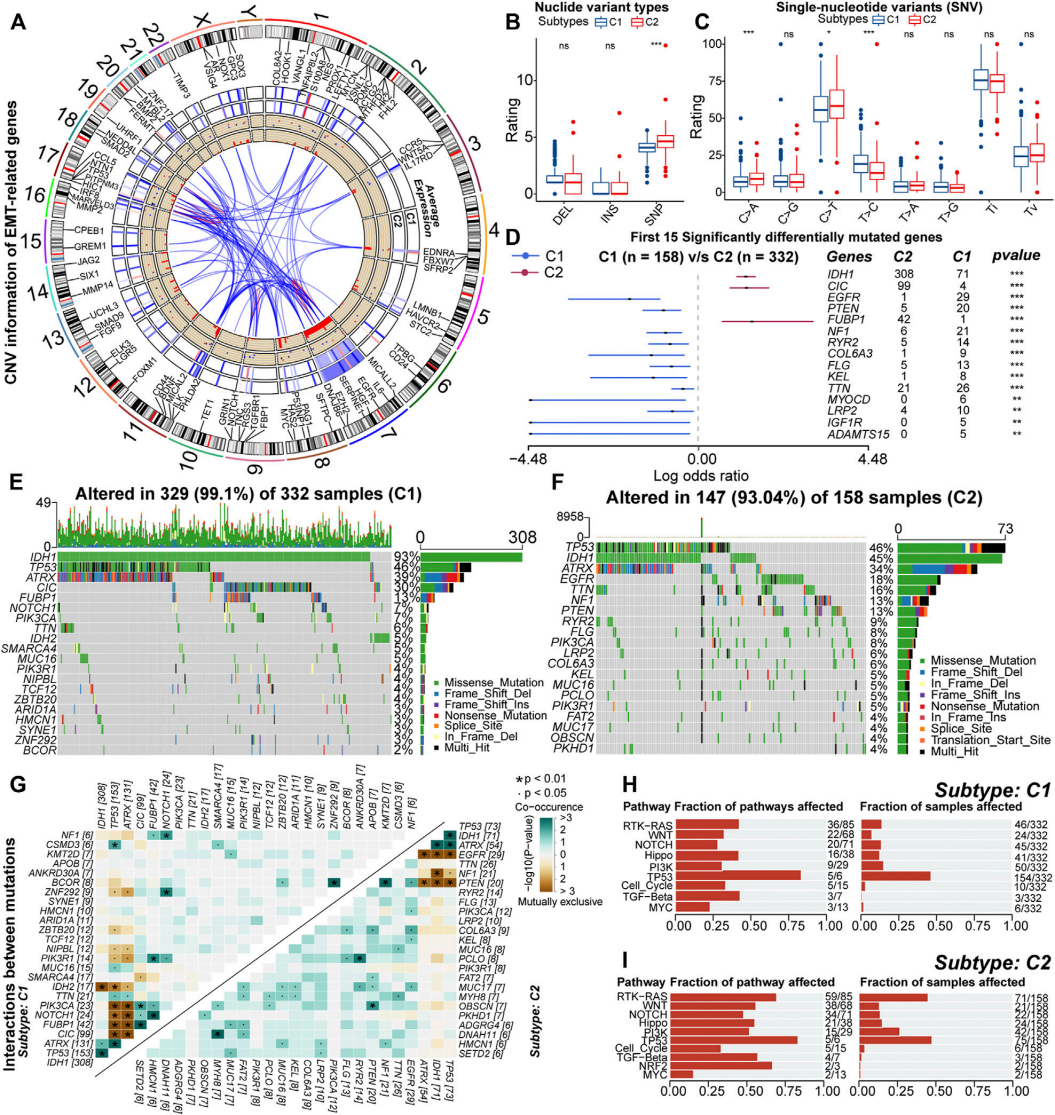
RCircos plot for copy number variations (CNV) of differential expressed EMT-related genes. From the outside to the inside, these loops represented chromosomal loci, average expression in samples with C1, average expression in samples with C2, gain (red) or loss (blue), and frequency of CNV, respectively **(A)**. Differences in the rating of deletion (DEL), insertion (INS), single-nucleotide polymorphism (SNP) between samples with C1 and C2 **(B)**. Differences in the rating of six types of single-nucleotide variants (SNV) including transitions (Ti) and transversions (Tv) between high and low GILncSig subgroups **(C)**. Differences in the mutation rates of the top 15 mutated genes in LGG between samples with C1 and C2 **(D)**. Waterfall plots of the 20 most frequently mutated genes in samples with C1 **(E)** and C2 **(F)**. The correlation heatmaps about correlations of 30 top mutated genes between high and low GILncSig subgroups. The color and symbol in each square represented the statistical significance of the exclusive or co-occurrence for each pair of genes, and the statistical method was the Fisher’s exact test **(G)**. The fraction of cancer signaling pathways and samples affected by the genetic variations in samples with C1 **(H)** and C2 **(I)**. In the box plots, *p* < 0.05 was indicated by “*”, *p* < 0.01 was indicated by “**”, *p* < 0.001 was indicated by “***”, and the statistical analysis was performed by the Mann-Whitney *U* test.

Further, we explored the variation in genetic features across the C1 and C2 subtypes. Of 123 EMT-related genes, 103 underwent CNV events (Supplementary Figure S5A). Further, we found 86 genes with significant amplifications or deletions. Some of these genes were reciprocally related and differentially expressed between samples with C1 and C2 (Figure 5A). In addition, Chr19/20 co-gain and Chr7 gain & Chr10 loss were almost only observed in samples with C2 (Supplementary Figures S5B,C). Also, the incidence of CDKN2A/B homozygous deletion, EGFR amplification, and PDGFRA amplification was higher in samples with C2 compared to C1 (Supplementary Figures S5D–G). Previous studies have indicated that these specific CNV events are related to high-grade gliomas and poor prognosis (Ceccarelli et al., 2016; Eskilsson et al., 2018; Reinhardt et al., 2018; Neftel et al., 2019). Therefore, variations in abnormal degrees of CNV events might existed between samples with C1 and C2, which might be involved in the altered expression patterns of EMT-related genes and formation of poor prognosis in samples with C2.

As for nucleotide variations, the median somatic mutation count in samples with C2 was 40, significantly higher than 27 in C1 (Supplementary Figure S5H). Also, for the variant types, the fraction of SNP was higher in samples with C2 (Figure 5B). In the six types of SNV, the proportion of C > T and C > A was higher in samples with C2, while the proportion of T > C was relatively lower (Figure 5C). Also, SNP and the missense mutation were predominant in both C1 and C2 subtypes (Supplementary Figures S5I,J). These results indicated that the variant types were not converted between samples with C1 and C2, but it was undeniable that genetic heterogeneity existed. For example, the mutation sites and forms of IDH1 were conserved, but it was highly mutated in samples with C1 (Supplementary Figure S5K). Also, in some genes such as TP53, CIC, and ATRX, the mutation sites and forms were different (Supplementary Figures S5L–N). In addition, VAF levels of samples with C2 were relatively higher (Supplementary Figure S5O). Notably, some studies have suggested that alterations in VAF levels are related to poor prognosis (Shin et al., 2017). In summary, nucleotide variations existed between samples with C1 and C2, which might affect the prognosis of these samples.

Next, by comparing the mutation profiles of samples with C1 and C2, a total of 261 differentially mutated genes were identified (Supplementary Table 5). Figure 5D exhibited the distribution of 15 most significantly differentially mutated genes. Among genes with mutation rates above 10%, IDH1, CIC, and FUBP1 were highly mutated in samples with C1, while EGFR, TTN, NF1, and PTEN were highly mutated in samples with C2 (Figures 5E,F). Previous studies have indicated that IDH1 mutations are protective factors for the prognosis of glioma, and mutations in CIC and FUBP1 are characteristic of oligodendroglioma (Yan et al., 2009; Wesseling et al., 2015). The EGFR mutation is one of the important markers of GBM and is associated with poor prognosis (Schlegel et al., 1994; Korshunov et al., 2015). In addition, PTEN and NF1 are oncogenes whose mutational inactivation is associated with cancer development and progression (Chalhoub and Baker, 2009; Krauthammer et al., 2015). As for driver genes, IDH1 was the driver gene for both samples with C1 and C2 (Supplementary Figures S5P,Q). This might be because IDH1 mutation is one of the key events in the oncogenesis of gliomas. In addition, EGFR was identified as the driver gene for samples with C2 (Figure 5E). The function of EGFR is closely related to the EMT process. Next, we analyzed the mutational interactions between the top 25 mutated genes. As shown in Figure 5G, most gene pairs exhibited mutually exclusive mutations in samples with C1, while gene pairs in samples with C2 mainly displayed co-occurring mutations. Interestingly, EGFR exhibited mutually exclusive mutations with IDH1, ATRX, and TP53. However, the mutation rates of ATRX and IDH1 were relatively lower in samples with C2 (Figures 5E,F). Finally, functional annotation was performed for differences in mutational patterns. Except for MYC and TP53, the remaining cancer signaling pathways were highly enriched in samples with C2 (Figures 5H,I). Among them, the WNT, NOTCH, PI3K, and TGF-β signaling pathways are closely related to EMT, and the RTK-RAS and PI3K signaling pathways can regulate cancer progression (Gonzalez and Medici, 2014; Katoh and Nakagama, 2014; Paul and Hristova, 2019; Hoxhaj and Manning, 2020). In addition, the Hippo and WNT signaling pathways are associated with the acquisition of stem cell features, and the activation of the NOTCH pathway can contribute to the formation of the immunosuppressive microenvironment (Zhan et al., 2017; Meurette and Mehlen, 2018; Ma et al., 2019). Taken together, genetic mechanisms broadly influenced the malignant progression of LGG. Also, differences in the activity of cancer signaling pathways might be related to the variations in multiple biological features including EMT levels, anti-tumor immunity, and stem cell features between samples with C1 and C2.

### 3.6 Epigenetic Variations Between Samples With Different Subtypes

**FIGURE 6:**
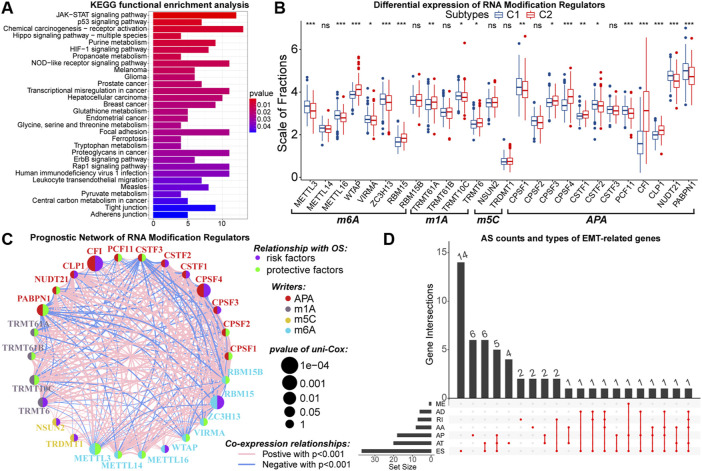
Results of KEGG functional enrichment analysis of DNA methylation driver genes **(A)**. Differences in the expression of N6-methyladenosine (m6A), N1-methyladenosine (m1A), 5-methylcytosine (m5C), and alternative polyadenylation (APA) writers between samples with C1 and C2 **(B)**. The interactive network and prognostic implications of writers for the post-transcriptional RNA modification. The left half-circle represented different RNA modification patterns, and the right half-circle represented whether these writers were risk or protective factors for OS. The colors of lines indicated correlations between writers, and the size of the circle represented pvalues for prognostic implications of these writers **(C)**. Statistics on the counts and types of genes with alternative splicing (AS) **(D)**. In the box plots, *p* < 0.05 was indicated by “*”, *p* < 0.01 was indicated by “**”, *p* < 0.001 was indicated by “***”, and the statistical analysis was performed by the Mann-Whitney *U* test.

Similarly, epigenetic derangements can drive the process of oncogenesis and progression through multiple approaches (Feinberg et al., 2016). As the most important pre-transcriptional epigenetic regulatory mechanism, DNA methylation can directly regulate gene expression. Between samples with C1 and C2, the expression of 617 genes was influenced by the DNA methylation levels. These genes were identified as driver genes of DNA methylation (Supplementary Table 6). Also, we found that the overall DNA methylation levels of driver genes in samples with C2 were relatively low (Supplementary Figure S6A). Previous studies have indicated that hypomethylation is associated with the oncogenesis process (Good et al., 2018). In addition, driver genes were highly enriched in KEGG pathways like glioma, cell metabolism, cell adhesion, and multiple cancer signaling pathways (Figure 6A). Therefore, altered DNA methylation patterns might be closely related to higher malignancy in samples with C2.

Further, we explored the potential link between EMT and post-transcriptional RNA regulatory patterns. From published data, a total of 26 writers for N6-methyladenosine (m6A), N1-methyladenosine (m1A), 5-methylcytosine (m5C), and alternative polyadenylation (APA) were selected (Huang et al., 2012; He et al., 2019; Shi et al., 2020). These writers were differentially expressed between samples with C1 and C2 (Figure 6B). Next, with cor > 0.5 and *p* < 0.05 as the threshold, 385 DEGs were identified to have co-expression relationships with these writers (Supplementary Table 7). Among them, 328 DEGs upregulated in samples with C2 were enriched in GO pathways related to the function of multiple immune molecules and cells (Supplementary Figure S6B). Moreover, these 328 DEGs were not only enriched in KEGG cancer signaling pathways such as PI3K-Akt, PD-1/PD-L1, and NF-kappa B but also could regulate multiple cell death patterns (Supplementary Figure S6C). In contrast, 57 DEGs lowly expressed in samples with C2 were relevant to the biological function of normal neurons (Supplementary Figures S6D,E). Notably, the expression of CFI was much higher in samples with C2, and 313 of 385 DEGs had co-expression relationships with CFI. Also, CFI was a prognostic risk factor in LGG samples (Figure 6C). Therefore, we considered that CFI might be an important APA regulator, whose hyper-expression was associated with the altered expression patterns of EMT-related genes and poor prognosis of LGG patients. In addition, we observed that 54 differentially expressed EMT-related genes had AS events (Figure 6D). In summary, the expression patterns of EMT-related genes were closely related to altered RNA modifications, which had a non-negligible impact on the malignant progression of LGG.

### 3.7 Construction of EMTsig and the Nomogram

**FIGURE 7:**
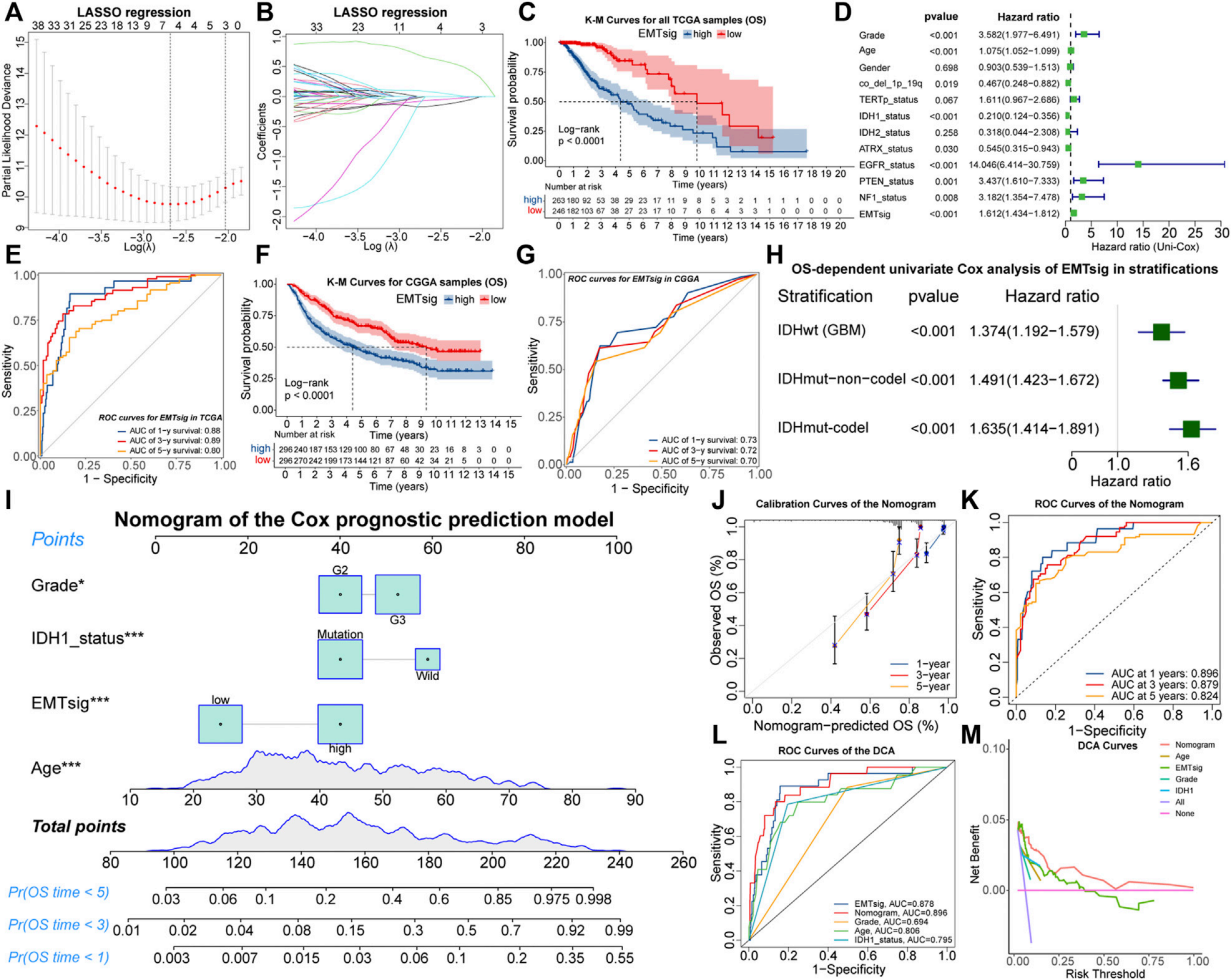
The relationship between lambda values and partial likelihood deviance **(A)** or variable coefficients **(B)** in the calculation of EMTsig by the LASSO regression. K-M curves of OS between the high and low EMTsig subgroups of the TCGA cohort **(C)**. Results of univariate Cox analysis of EMTsig, clinical traits, and molecular traits with OS in LGG patients of the TCGA cohort **(D)**. The 1-, 3-, and 5-year OS-dependent ROC curves of EMTsig in the TCGA cohort **(E)**. K-M curves of OS between the high and low EMTsig subgroups of the CGGA cohort **(F)**. The 1-, 3-, and 5-year OS-dependent ROC curves of EMTsig in the CGGA cohort **(G)**. Results of univariate Cox analysis of EMTsig with OS in three stratifications with worse prognosis **(H)**. The nomogram of the Cox prognostic prediction model **(I)**. Calibration curves **(J)** and OS-dependent ROC curves **(K)** for the nomogram. The OS-dependent ROC curves for clinical traits, IDH1status, the nomogram, and EMTsig **(L)**. Decision curves compared the differences in the net benefit of clinical traits, IDH1status, EMTsig, and the nomogram for the prognostic prediction of LGG patients **(M)**. In this figure, *p* < 0.05 was indicated by “*”, *p* < 0.01 was indicated by “**”, *p* < 0.001 was indicated by “***”.

To simplify the forecasting process and quantify the prognostic implications of EMT-related genes, all 509 LGG samples in the TCGA cohort were randomly divided into the train (*n* = 256) and test (*n* = 253) sets, of which there were no differences in the distribution of clinical traits (Supplementary Table 8). Next, we determined the optimal penalty coefficient (log(λ) = −2.7) by LASSO regression, and BMP2, SFRP2, BIRC5, and ZNF217 were identified as characteristic variables of EMT-related genes (Supplementary Table 9; Figures 7A,B). After testing by the univariate and multivariate Cox analysis, all of these four genes retained prognostic significance (Supplementary Figures S7A,B). Therefore, EMTsig was constructed based on them. The K-M curves indicated that high EMTsig accompanied by worse prognosis in LGG samples (Figure 7C; Supplementary Figures S7C,D). Also, the results of univariate Cox analysis presented that the hazard ratio of EMTsig was 1.612 (1.434–1.812, *p* < 0.001) (Figure 7D). Thus, EMTsig was a prognostic risk factor for LGG.

We further validated the accuracy and versatility of the prognostic predictive capability of EMTsig. The OS-dependent ROC curves indicated that the AUC values corresponding to 1-, 3-, and 5-year OS were 0.88, 0.89, and 0.80 (Figure 7E). Similarly, the high EMTsig subgroup of CGGA samples also had a poor prognosis (Figure 7F). As for the ROC curves, the AUC values of EMTsig corresponding to 1-, 3-, and 5-year OS in CGGA were all above 0.7 (Figure 7G). In addition, the levels of IDH mutation and 1p19q co-deletion levels in the low EMTsig subgroup of CGGA samples were higher, further confirming that elevated EMTsig was accompanied by the increase of malignancy (Supplementary Figures S7E,F). Notably, 470 of 509 TCGA samples were obtained from Caucasians, while the CGGA samples were obtained from Chinese (Asians). Therefore, EMTsig could be a reliable prognostic biomarker in patients from different sources or ethnicities.

The result of multivariate Cox analysis indicated that the prognostic prediction capability of EMTsig was independent of clinical and molecular traits which might have a significant effect on the prognosis of LGG (Supplementary Figure S7G). Next, the TCGA and CGGA cohorts were further stratified. Firstly, we stratified all LGG samples based on clinical traits. K-M curves indicated that the low-EMTsig subgroup had a better prognosis in each clinical trait stratification (Supplementary Figures S7H–M). Secondly, the CNS WHO 2021 classification integrates the prognostic implications of IDH mutations and 1p19q-codel and classifies adult-type diffuse gliomas into three groups (Louis et al., 2021). Following the same strategy, we identified three stratifications, including 470 samples with IDHwt (GBM), 431 samples with IDHmut-non-codel (Astrocytoma), and 254 samples with IDHmut-codel (Oligodendroglioma). By univariate Cox analysis, we found that EMTsig was a significant prognostic risk factor in all of these three stratifications (Figure 7H). These results further supported that EMTsig could maintain the significant prognostic predictive capability in glioma patients of different clinical and molecular trait stratifications. Also, based on the properties of EMTsig as an independent prognostic biomarker, we considered whether a comprehensive assessment of EMTsig, clinical traits, and molecular traits could maximize the survival benefit of LGG patients in clinical applications. With the Cox regression, we found that IDH1 mutation status, age, grade, and EMTsig had independent effects on OS, and the nomogram was constructed (Figure 7I). The calibration and ROC curves exhibited that the error of the nomogram was within a manageable range (Figures 7J,K). Moreover, the nomogram had the highest AUC value compared to the single-trait evaluation approaches (Figure 7L). Also, through decision curve analysis (DCA), the nomogram had the highest net benefit (Figure 7M). In summary, in terms of prognostic predictive capability, the nomogram could be more sophisticated compared to the EMTsig.

### 3.8 Guidance of EMTsig on Biological Properties of LGG

**FIGURE 8:**
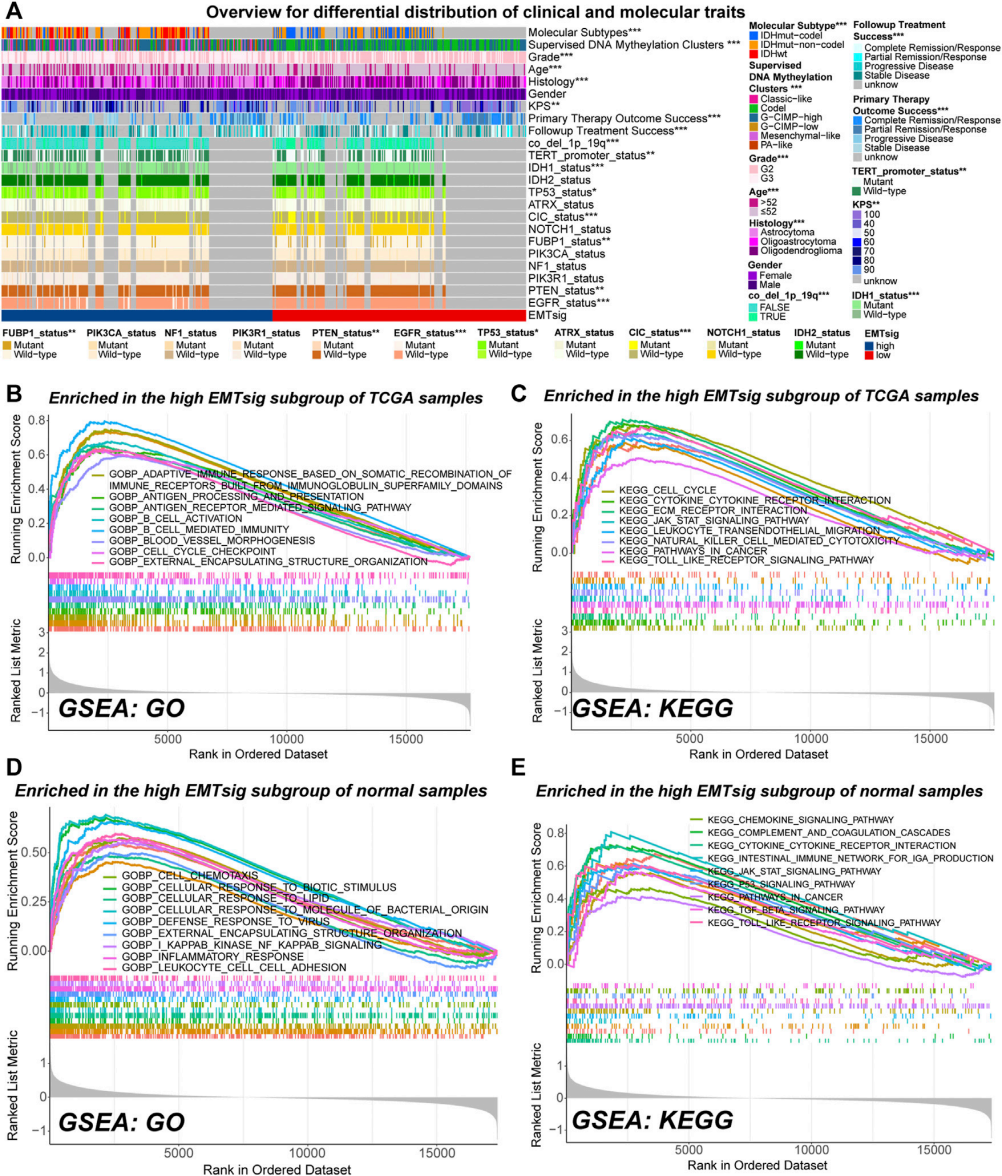
Overview of differences in clinical traits and molecular traits between the high and low EMTsig subgroups of LGG samples in the TCGA cohort **(A)**. Gene set enrichment analysis (GSVA) results of activated GO **(B)** and KEGG **(C)** pathways in the high EMTsig subgroup of TCGA samples. GSVA results of activated GO **(D)** and KEGG **(E)** pathways in the low EMTsig subgroup of normal samples. In this figure, *p* < 0.05 was indicated by “*”, *p* < 0.01 was indicated by “**”, *p* < 0.001 was indicated by “***”.

Since EMTsig was constructed based on the principle components of 121 differentially expressed EMT-related genes, EMTsig might be correlated with EMT-related subtypes. As shown in Supplementary Figure S8A, EMTsig was significantly higher in samples with C2. Also, 95% of samples with C2 were in the high EMTsig subgroup (Supplementary Figure S8B). Figure 8A provided an overview of the differences in clinical and molecular traits between high and low EMTsig subgroups of all LGG samples in the TCGA cohort. The high EMTsig subgroup had similar clinical and molecular traits as C2. Also, EMTsig was positively correlated with ESTIMATE score and mDNAsi, and negatively correlated with mRNAsi (Supplementary Figures S8C–E). Thus, samples in the high EMTsig subgroup might possess similar biological properties as C2. Encouragingly, EMTsig was an easily detectable quantitative biomarker. Therefore, EMTsig could assist in identifying the biological properties of LGG patients in clinical applications, which facilitates the selection of appropriate individualized interventions.

GSVA can be used to detect changes in the activity of pathways within the entire gene set. In both normal and LGG samples in the TCGA cohort, most of the highly activated gene sets in the low EMTsig subgroup were just related to the transduction of neural signals (Supplementary Figures S8F–I). In contrast, immune function, cell cycle, EMT, and cancer-related signaling pathways were highly activated in the high-EMTsig subgroup of LGG samples (Figures 8B,C). Notably, similar results were obtained in the high EMTsig subgroup of normal samples (Figures 8D,E). This indicated that adjacent non-tumor tissue might also participate in the EMT process, formation of TME, and malignant progression of LGG. Overall, EMTsig was suggestive for the evolving trend of biological properties of LGG. Also, the impact of adjacent non-tumor tissue should be considered when selecting therapeutic strategies for patients with high EMTsig in clinical applications.

### 3.9 Guidance of EMTsig on the Sensitivity of Oncology Treatment

Many studies have pointed out that the EMT process and accompanying alterations in immune or stemness features could impact the efficiency of oncology treatment (Mani et al., 2008; Zhou et al., 2017; Singh et al., 2018; van Staalduinen et al., 2018; Ramesh et al., 2020). Based on the suggestive capability of EMTsig for EMT levels and biological properties, EMTsig might be a potential biomarker for the sensitivity of oncology treatment in LGG.

**FIGURE 9:**
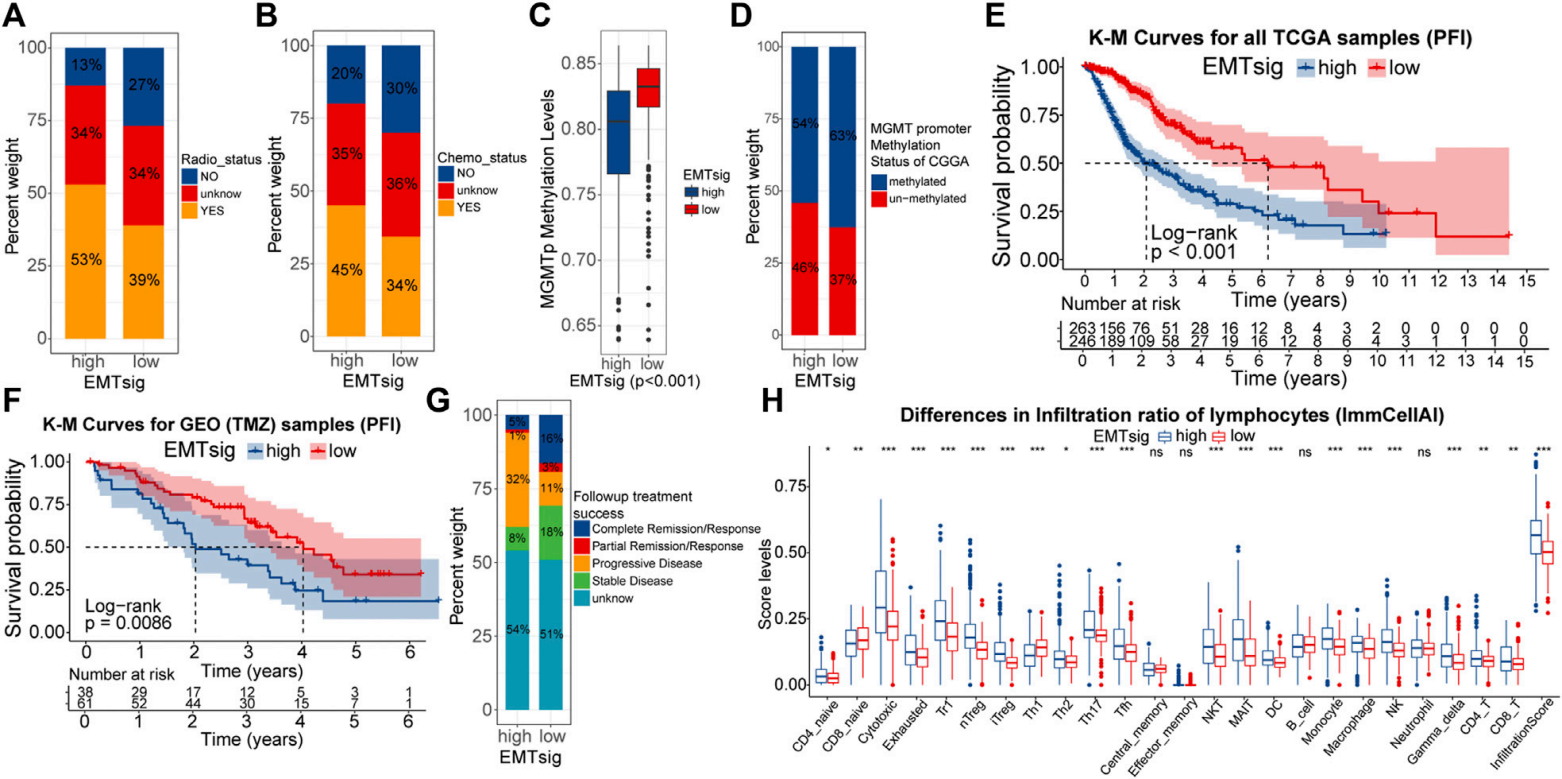
Differences in the sensitivity of radiotherapy **(A)** and chemotherapy **(B)** between high and low EMTsig subgroups. Differences in the MGMT promoter (MGMTp) methylation levels between high and low EMTsig subgroups in the TCGA cohort **(C)**. Differences in the MGMT promoter (MGMTp) methylation status between high and low EMTsig subgroups in the CGGA cohort **(D)**. K-M curves of Progression-free interval (PFI) between high and low EMTsig subgroups of the TCGA cohort **(E)** and the GEO cohort **(F)**. Differences in follow-up treatment success between high and low EMTsig subgroups of TCGA samples **(G)**. Differences in the lymphocyte infiltration levels (ImmuCellAI) between the high and low EMTsig subgroups of the TCGA cohort **(H)**. In this figure, *p* < 0.05 was indicated by “*”, *p* < 0.01 was indicated by “**”, *p* < 0.001 was indicated by “***”. Variance analyses were performed with the Mann-Whitney *U* test.

Firstly, we verified the relationship between EMTsig and efficiency to radiotherapy and chemotherapy. In the TCGA cohort, the proportion of samples who were sensitive to radiotherapy and chemotherapy was higher in the high EMTsig subgroup (Figures 9A,B). Also, we noted that the MGMT promoter (MGMTp) methylation levels were higher in the high EMTsig subgroup (Figure 9C). Since glioma chemotherapy is mainly dependent on the alkylating agent TMZ, the high MGMTp methylation level further supported that the high EMTsig subgroup was sensitive to TMZ. Meanwhile, in the CGGA cohort, we obtained similar results (Figure 9D). Further, we analyzed the relationship between the progression-free interval (PFI) and EMTsig. However, in the TCGA cohort, K-M curves indicated that the high EMTsig subgroup had a lower PFI time and rate (Figure 9E). Likewise, in a GEO cohort that received TMZ therapy, elevated EMTsig also adversely affected the PFI (Figure 9F). Moreover, in the TCGA cohort, 32% of samples in the high EMTsig subgroup remained in progressive disease after follow-up treatment, this proportion was only 11% in samples with low EMTsig. Also, the probability of achieving complete remission was higher in samples in the low EMTsig subgroup (Figure 9G). These results indicated that although the rise in EMTsig could improve the sensitivity of radiotherapy and TMZ chemotherapy, the concomitant elevated malignancy might neutralize the therapeutic efficiency, and thus the long-term outcome was not satisfactory. Finally, in the CellMiner database, we explored possible alternative chemotherapeutic agents. We found that the sensitivity of some chemotherapeutic agents and molecular targeted drugs were correlated with the expression of EMTsig-related genes (Supplementary Figures S9A,B, Supplementary Table 10). Although these drugs are not widely used in the chemotherapy of glioma, but they provide potential alternative individualized therapeutic plans for LGG.

The essence of ICB therapy is the reactivation of suppressed anti-tumor immune responses. Tumor mutational load (TMB) is closely related to the formation of neoantigenic epitopes, which can be used as indices for the sensitivity of ICB therapy (Chan et al., 2019). Supplementary Figures S9C demonstrated that TMB was positively correlated with EMTsig. Also, the proportion of immune cells, especially lymphocytes, is closely related to the sensitivity of ICB therapy (Neal et al., 2018; Chan et al., 2019). Through the ImmuCellAI algorithm, the infiltration scores of 24 types of immune cells were obtained for TCGA samples (Supplementary Table 11). As shown in Figure 9H, the proportion of most lymphocytes and the infiltration score were significantly higher in the high EMTsig subgroup. Meanwhile, the ESTIMATE score was positively correlated with EMTsig, indicating that samples in the high EMTsig subgroup had lower tumor purity (Supplementary Figure S8C). In addition, immune checkpoints were highly expressed in the high EMTsig subgroup with higher dysfunction scores (Supplementary Figures S9D,E). This implied that the highly expressed immune checkpoints might inhibit the normal function of lymphocytes. However, highly expressed immune checkpoints provided adequate sites for ICB therapy, and highly infiltrated lymphocytes supplied potential effector cells for the recovery of anti-tumor immune responses. Therefore, EMTsig might be positively correlated with the sensitivity of ICB therapy for LGG patients.

## 4 Discussion

Our study aimed to identify EMT-related genes in that have a significant impact on prognosis and explore the relationship of these genes with oncogenesis, progression, and the evolution of biological properties in LGG. By optimizing the clustering strategy, two subtypes about differential expression patterns of EMT-related genes were identified in the TCGA cohort. Between the C1 and C2 subtypes, significant differences existed in clinical traits, molecular traits, metabolism, anti-tumor immunity, and stemness features, eventually resulting in poor prognosis of samples with C2. Also, we found that genetic, epigenetic, and transcriptomic heterogeneity led to variations in the activation of tumor signaling pathways and molecular functions, thus causing the differential distribution of malignant features. Next, to better evaluate individual variations between LGG samples, the EMTsig system was constructed. As expected, EMTsig was not only a prognostic marker but also correlated with the sensitivity of oncology treatment. In addition, EMTsig provided us with an easily conductible clustering strategy for evaluating the role of adjacent non-tumor tissue in the malignant progression of LGG. The GSVA results indicated that adjacent non-tumor tissue might also be involved in the process of EMT, progression, metastasis, and formation of the TME in LGG. Therefore, when performing clinical interventions, EMTsig allowed us for evaluating the features of the tumor and adjacent non-tumor tissue. This could be beneficial for LGG patients to establish the optimal individualized therapeutic plans for improving clinical outcomes.

In the context of neoplasia, the EMT process is often aberrantly activated. EMT can assist in metastasis of cancer cells by enhancing motility, disrupting intercellular junctions, degrading the basement membrane, and remodeling the ECM (Miyoshi et al., 2004; Costigliola et al., 2017; Loh et al., 2019). Also, the EMT process is associated with the rise in the proportion of cancer stem cells, decreased anti-tumor immunity, and resistance to oncology treatment (Rhim et al., 2012; Akalay et al., 2013; Singh et al., 2018; van Staalduinen et al., 2018). Thus, EMT is one of the essential parts of the invasion-metastasis cascade. Although the heterogeneity exists between different cancer types, the EMT process in LGG may also possess significant prognostic implications. To further explore this issue, we identified the EMT-related genes most significantly differentially expressed between LGG and normal samples and provided a comprehensive insight into the mechanisms by which these genes affected the evolution of biological features, oncogenesis, progression, and prognosis. Our study indicated that the expression of EMT-TFs, EMT-related signaling pathways, and the ECM process were closely related to the expression patterns of 121 differential expressed EMT-related genes. This suggested that our subtypes could well reflect the differences in EMT levels between LGG samples. Meanwhile, we noted that samples with C2 had lower tumor purity, higher expression of immune checkpoints, and a higher proportion of pro-tumor cells such as Tregs, M2 macrophages, and resting immune cells. This resulted in higher levels of T-cell exhaustion and poorer anti-tumor immunity. In fact, several studies found that some LGG samples in the TCGA cohort had immunological characteristics similar to our C2 subtype, which possessed a poorer prognosis (Du et al., 2020; Wu et al., 2020; Ji et al., 2021). Also, samples with C2 were more likely to acquire higher stemness levels. These altered biological properties would have a profound impact on the clinical outcome of LGG patients. In addition, previous studies have noted that stromal cells in tumor tissue can secrete a variety of chemokines and cytokines. These secreted signals may communicate with tumor cells, immune cells, or adjacent non-tumor tissue via the paracrine fashion (Olumi et al., 1999; Quail and Joyce, 2013; Sun et al., 2014; Lambrechts et al., 2018). Similarly, we observed that the adjacent non-tumor tissue appeared to be adaptively altered in response to the changes in EMTsig. Therefore, the permissive effect of adjacent non-tumor tissue on the invasion-metastasis cascade should be considered when implementing clinical interventions for LGG patients.

We noted that differences in EMT levels, molecular traits, and genetic features were simultaneously observed between samples with C1 and C2. Similarly, various studies have pointed out that EMT can cause genetic variations through a variety of mechanisms. For example, TGF-β can inhibit DNA damage repair and prolong mitosis (Comaills et al., 2016). Also, TGF-β and SNAI1 can affect the integrity of nuclear envelope and nuclear pore components, leading to abnormal mitotic processes and decreased mechanical stability of the nucleus (Güttinger et al., 2009; Gruenbaum and Foisner, 2015). These alterations lead to prolonged exposure of DNA in the cytoplasm and increased risk of nuclear deformation or rupture when cells undergo strenuous movement, eventually leading to the accumulation of genomic instability. In our study, samples with C2 were more inclined to possess mutated EGFR, TTN, NF1, and PTEN. Mutations of these genes are often indicated for higher degrees of malignancy and poor clinical outcomes. Also, multiple cancer signaling pathways were highly aberrantly activated in samples with C2. These signaling pathways are closely related to the EMT process, immune cell infiltration, cancer stem cell accumulation, progression, and metastasis of cancers. Therefore, our study offered a possibility that the EMT process might interact with genetic features, which could facilitate the malignant progression of LGG.

In contrast to genetic variations, epigenetic modifications regulate gene expression without changing DNA sequences or affecting protein functions at the translational level. Our study suggested that the variations in expression patterns of EMT-related genes were related to changes in DNA methylation and post-transcriptional RNA modification patterns in LGG. Many studies have pointed out that EMT-TFs can synergistically co-regulate the EMT process with DNA methylation. For example, CDH1 is an important marker of EMT and is often down-regulated or inactivated during the progression of cancers (Loh et al., 2019). In EMT-TFs, SNAI1 and SNAI2 bind to the E-box on the promoter and directly inhibit the transcription of CDH1 (Puisieux et al., 2014). Meanwhile, the CDH1 promoter exhibited hypermethylation in a variety of cancers, further reducing CDH1 expression (Yoshiura et al., 1995). Similarly, altered RNA regulation patterns may mediate the EMT process. For example, m6A in SNAI1 CDS triggers the polyribosome-mediated translation process which drives the activation of SNAI1-related functions (Lin et al., 2019). In addition, we observed that many EMT-related genes underwent AS events in LGG samples. Depending on the splice site, AS has different biological effects. In case it happens in the RNA coding regions, AS events can affect protein functions, while those occurring in the polyA site may impact the efficiency of translation (Keren et al., 2010). As the 3′UTR contains multiple polyA sites, this process may be affected by the APA activity. In our study, the expression of APA writers varied across different subtypes in LGG samples. Thus, we believed that the differences in epigenetic regulatory patterns were associated with differential expression patterns of EMT-related genes. These changes might be involved in the regulation of EMT and affected the biological properties and clinical outcomes of LGG patients.

Finally, we attempted to translate our findings into clinical applications. the EMTsig system successfully quantified the EMT levels and could reliably predict the prognosis and the evolutionary patterns of biological properties in LGG patients. Also, comparing the available evidence, we found that the four genes used to construct the EMTsig were closely related to EMT. BMP2 is a member of the TGFβ family and induces the process of EMT through the BMP signaling pathway (Fukuda et al., 2021). SFRP2 is an upstream repressor of the WNT signaling pathway and has an inhibitory effect on the EMT process (Duan et al., 2017). BIRC5 is one of the anti-apoptotic signals induced by ZEB1, which can induce the EMT process through the TGFβ signaling pathway (Sánchez-Tilló et al., 2014). ZNF217, a member of the zinc finger protein family, functions as an EMT inducer mainly through the TGF-β-activated SMAD signaling pathway (Vendrell et al., 2012). Therefore, EMTsig is closely related to the activity of the TGF-β signaling pathway, which means that EMTsig may accurately reflect changes in the biological processes associated with the TGF-β signaling pathway. In addition, EMTsig could help LGG patients to optimize the therapeutic strategies. Currently, TMZ is the main chemotherapeutic agent for glioma. Our study revealed that EMTsig was positively related to the sensitivity of TMZ. However, the increase in EMT levels and malignancy resulted in an unsatisfactory long-term outcome of TMZ chemotherapy. Similar evidence suggests that the efficacy of TMZ can be improved by inhibiting the EMT process, further supporting our findings (Rajesh et al., 2020). Moreover, the rise in EMTsig gave rise to several alternative therapeutic options. In our study, samples with high EMTsig had higher lymphocyte infiltration, immune checkpoint expression, and TMB, which indicated that ICB therapy might be effective. Similar studies have pointed out that the EMT process can activate the PD-L1 signaling to induce immune escape, and ICB therapy may be an alternative therapeutic option (Jiang and Zhan, 2020). Also, EMTsig-related genes were related to the sensitivity of some chemotherapeutic agents and molecular targeted drugs. Therefore, the individualized alternative chemotherapy protocols for LGG patients with TMZ resistance might be a good choice. In addition, therapeutic strategies for targeting key molecules and signaling pathways of EMT have promising efficacy in cancers (Davis et al., 2014; Zhu et al., 2018). In the future, cellular and animal model-based studies may further confirm the feasibility of therapeutic strategies for targeting the EMT process in LGG patients. In summary, EMTsig was valuable for clinical application and also provided us with new insights and approaches for investigating the effects of EMT on the biological properties of cancers.

There were still some limitations in this study. By using the differentially expressed EMT-related genes, we explored the impact of the EMT-related genes on the biological properties of LGG at the macroscopic level. However, The EMT-related genes we identified have other biological functions alongside the induction of EMT. This means that the differential expression of EMT-related genes cannot fully explain the interaction patterns between EMT levels and other LGG biological properties. Thus, the function and regulatory mechanism of specific genes need to be further elucidated. Further, the identification of EMTsig was based on existing data sets. However, the clinical significance of EMTsig was not validated with RT-qPCR in our study. In the future, this can be further explored in newly acquired clinical samples or disease models. In addition, the mechanisms behind the prognostic implications of adjacent non-tumor tissue in LGG need to be further validated in experimental studies. However, it is undeniable that our study provides new insights for LGG biology and oncology treatment, which has important implications for clinical interventions of LGG.

## 5 Conclusion

This work was based on a multi-omics analysis and revealed the mechanisms by which EMT-related genes affects the biological properties of LGG from the genetic, epigenetic, and transcriptomic layers. Meanwhile, by revealing changes in the activity of signaling pathways, we explored the potential biological functions of adjacent non-tumor tissue in EMT processes, oncogenesis, progression, and metastasis of LGG. Finally, EMTsig was a promising versatile biomarker that could accurately predict prognosis and guide oncology treatment for LGG patients. In the future, experimental studies can help us to more comprehensively understand the mechanisms behind the EMT process and to validate the prognostic implications in adjacent non-tumor tissue. Additionally, after being validated by prospective studies, EMTsig may be valuable for the individualized treatment of LGG.

### Data Availability Statement

The datasets presented in this study can be found in online repositories. The names of the repository/repositories and accession number(s) can be found in the article/Supplementary Material.
