# Supplementary material for: Identification of EMT-Related Genes and Prognostic Signature With Significant Implications on Biological Properties and Oncology Treatment of Lower Grade Gliomas
Source: Front Cell Dev Biol. 2022 May 17;10:887693. doi: 10.3389/fcell.2022.887693 (PMC9152435; doi:10.3389/fcell.2022.887693)

**Supplementary Figure S1:** Overview of our study design.

­­­


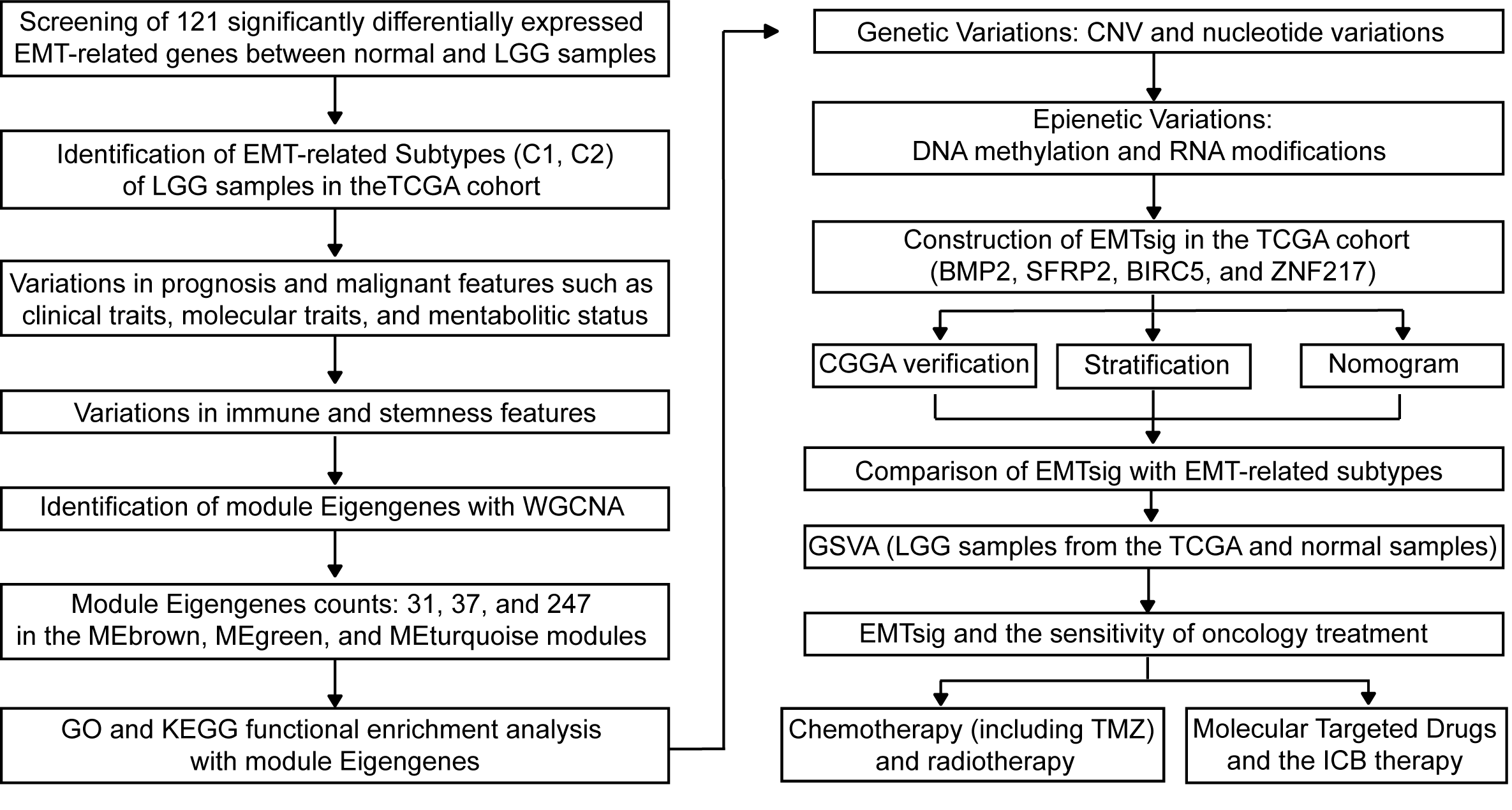


**Supplementary Figure S2:** NMF rank survey helps us to choose the appropriate rank value. The cophenetic and dispersion values varied most at 2-3, so the optimal rank value is 2 for LGG samples **(A)**. Differences in the expression of MMP2/3/9 **(C),** E-cadherin (CDH1) and N-cadherin (CDH2) **(B)**, vimentin (VIM) **(D)** between samples with C1 and C2. In the box plots, p<0.05 was indicated by "*”, p<0.01 was indicated by "**", p<0.001 was indicated by "***", and the statistical analysis was performed by the Mann-Whitney U test.


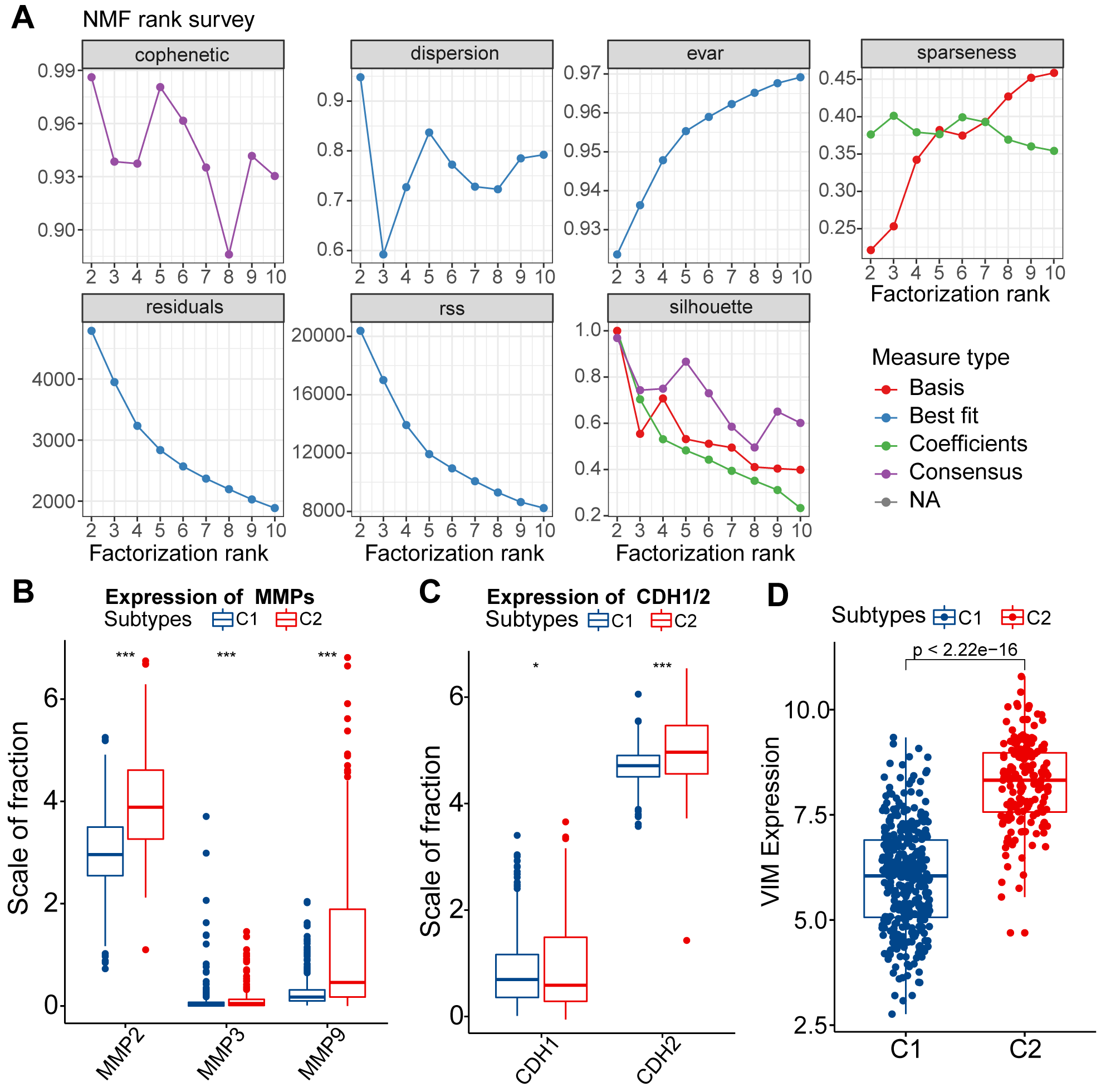


**Supplementary Figure S3:** Correlation heatmap of immune cell infiltration. The statistical method was the Spearman correlation analysis. Red and blue represented positive and negative correlations **(A)**. Differences in the immune scores **(B)**, stromal scores **(C)**, mDNAsi **(D)**, and mRNAsi**(E)** between samples with C1 and C2. K-M curves between samples with high and low (clustering method: median values) mDNAsi **(F)** and mRNAsi **(G)**. Variations in the ESTIMATE score between samples with C1 and C2 **(H)**. In the box plots, p<0.05 was indicated by "*”, p<0.01 was indicated by "**", p<0.001 was indicated by "***", and the statistical analysis was performed by the Mann-Whitney U test.


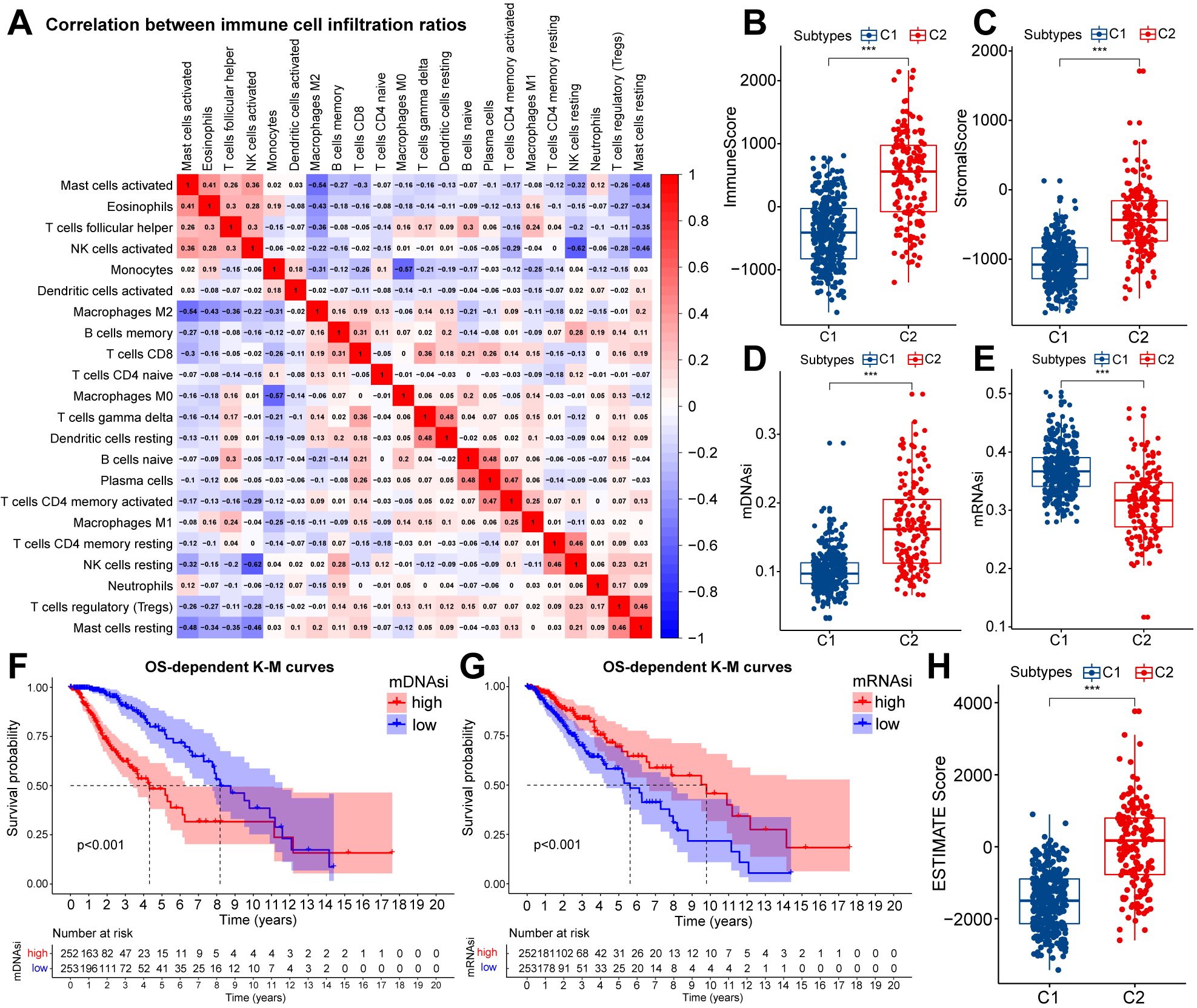


**Supplementary Figure S4:** Results of functional enrichment analysis containing: GO **(A)** and KEGG **(B)** enrichment analysis of Eigengenes in the MEturquoise module, GO **(C)** and KEGG **(D)** enrichment analysis of Eigengenes in the MEbrown module, and GO enrichment analysis of Eigengenes in the MEgreen module **(E)**.


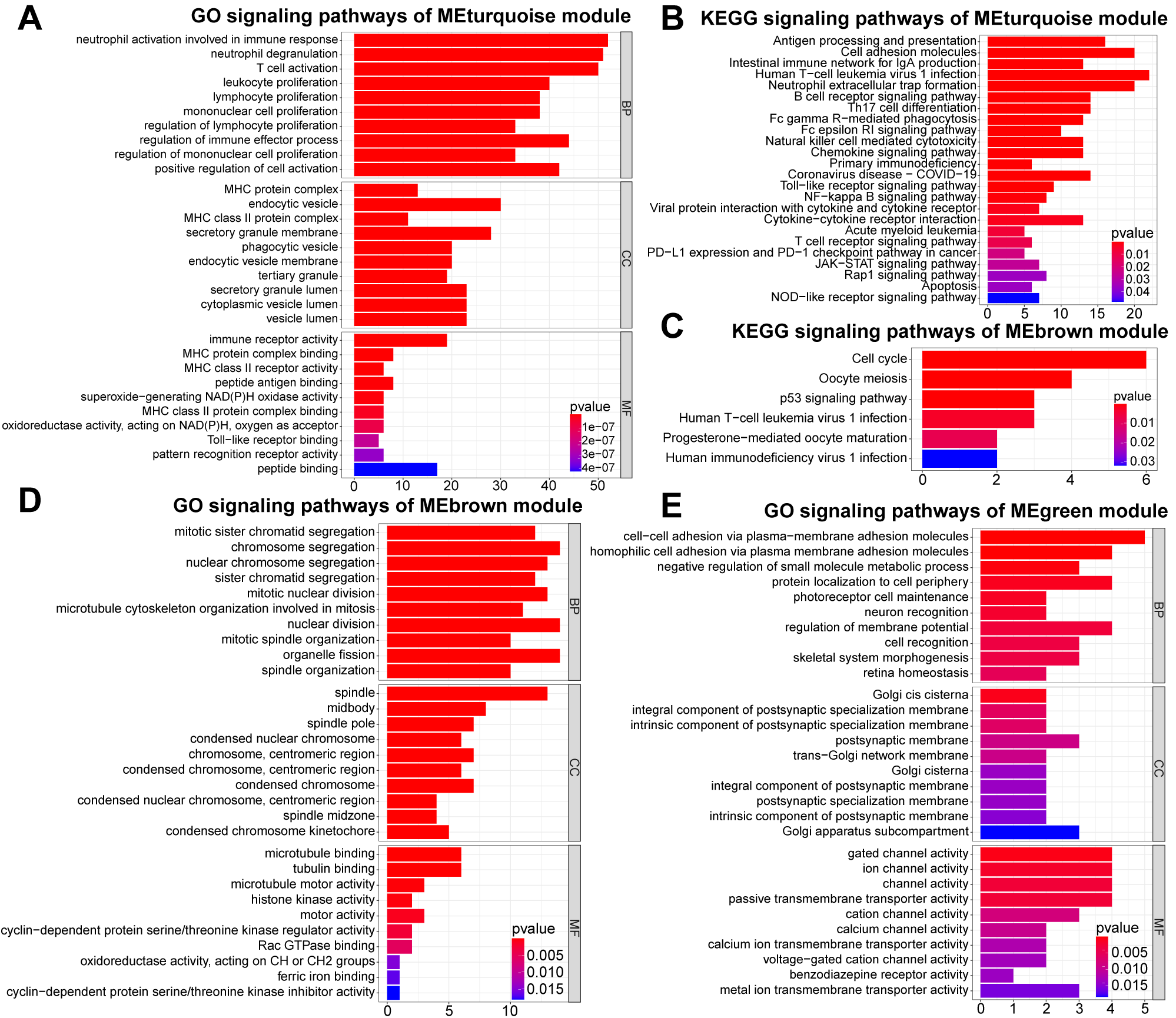


**Supplementary Figure S5:** Frequency distribution of GAIN versus LOSS for EMT-related genes with copy number variation (CNV) events **(A)**. Differential distribution of Chr7 gain & Chr10 loss **(B)**, Chr19/20 co-gain **(C)**, CDKN2A homozygous deletion **(D)**, CDKN2B CDKN2A homozygous deletion **(E)**, EGFR amplification **(F)**, and PDGFRA amplification **(G)** between samples with C1 and C2. Differences in the somatic mutation count between samples with C1 and C2 **(H)**. Overview of genetic variations of samples with C1 **(I)** and C2 **(J)**. The lollipop plots displayed the differential distribution of mutation sites, rates, and types for IDH1 **(K)**, CIC **(L)**, TP53 **(M)**, and ATRX **(N)**. The color of points represents the mutation type, and the position of points represents the mutation sites. Differences in the overall variant allele frequency (VAF) levels between samples with C1 and C2 **(O)**. Driver genes for samples with C1 **(P)** and C2 **(Q)**.


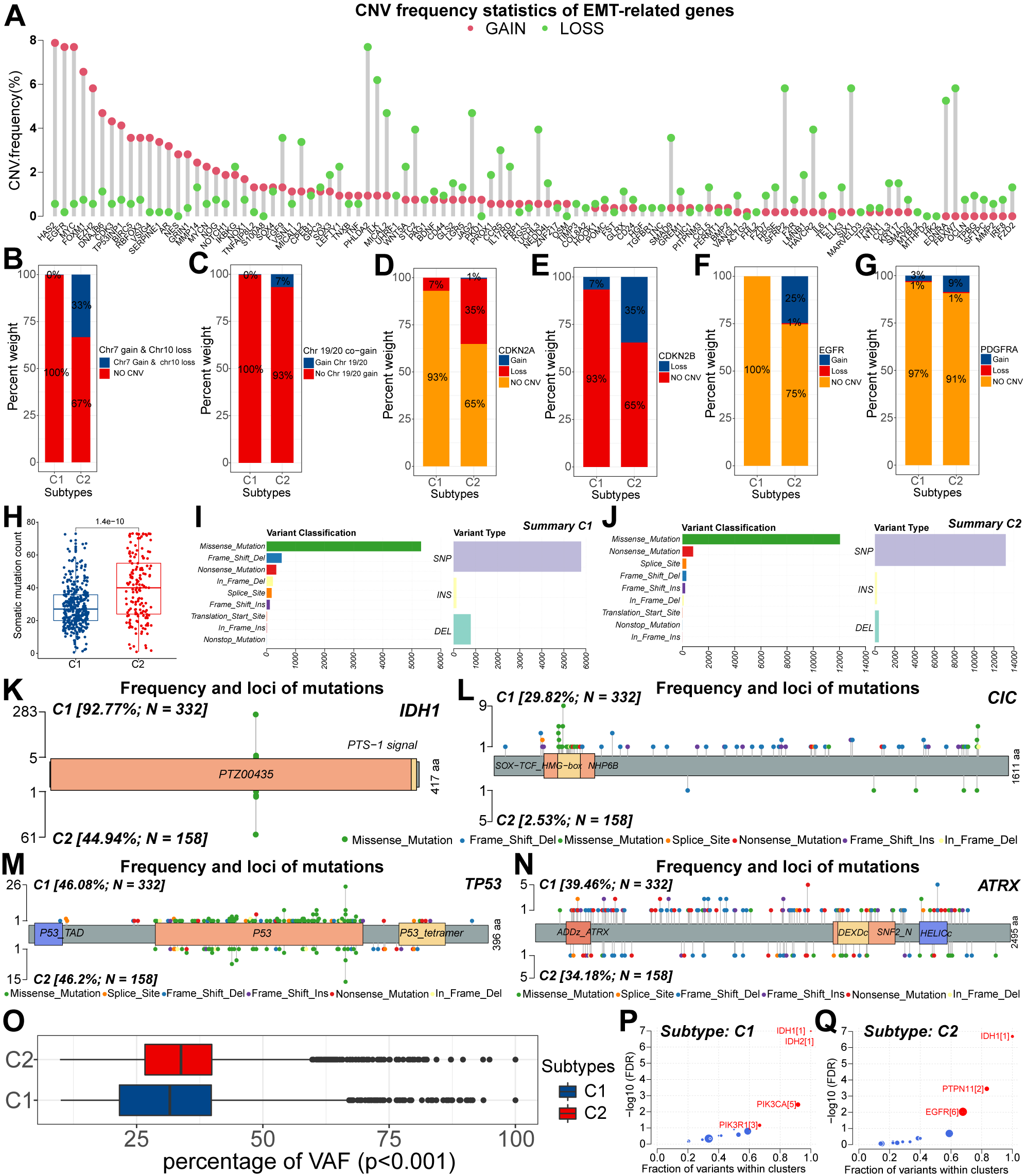


**Supplementary Figure S6:** The heat map presents the difference in methylation levels of DNA methylation driver genes between samples with C1 and C2 **(A)**. Results of GO **(B)** and KEGG **(C)** enrichment analysis of 328 up-regulated DEGs which were co-expressed with writers for the post-transcriptional RNA modification. Results of GO **(D)** and KEGG **(E)** enrichment analysis of 57 down-regulated DEGs which were co-expressed with writers for the post-transcriptional RNA modification.


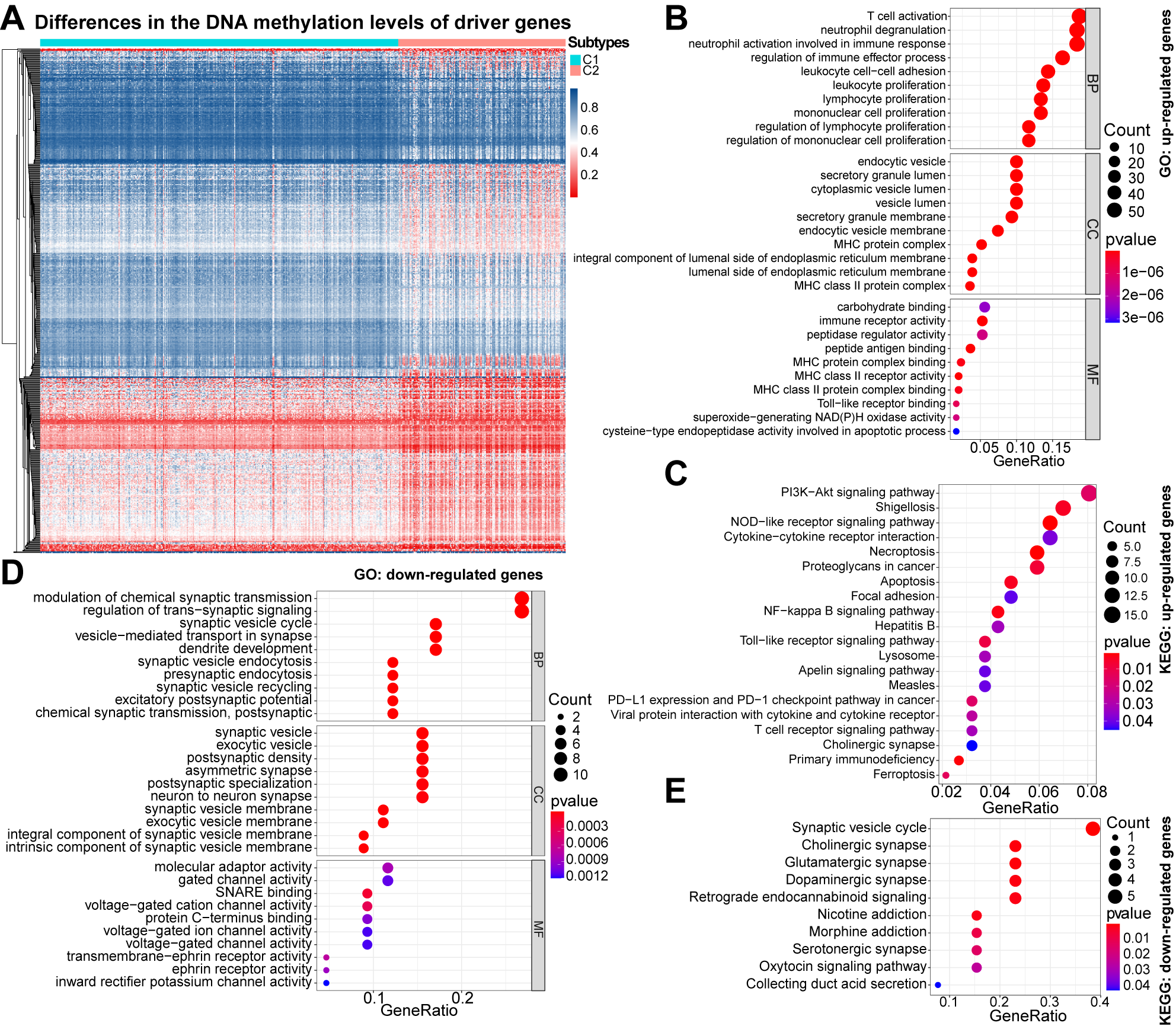


**Supplementary Figure S7:** Results of univariate **(A)** and multivariate **(B)** Cox analysis between the expression of EMTsig-related genes and OS in LGG patients. K-M curves of OS between high and low EMTsig subgroups of samples in the train **(C)** and test **(D)** sets. Differences in the composition ratios of the IDH mutation **(E)** and 1p19q co-deletion **(F)** statuses between the high and low EMTsig subgroups of LGG samples in the CGGA cohort. The result of multivariate Cox analysis of EMTsig, clinical traits, and molecular traits with OS in LGG patients of the TCGA cohort **(G)**. Stratified survival analysis for OS and EMTsig, including six stratifications: senior (Age>52) **(H)**, junior (Age≤52) **(I)**, female **(J)**, male **(K)**, G2 **(L)**, and G3 **(M)**.


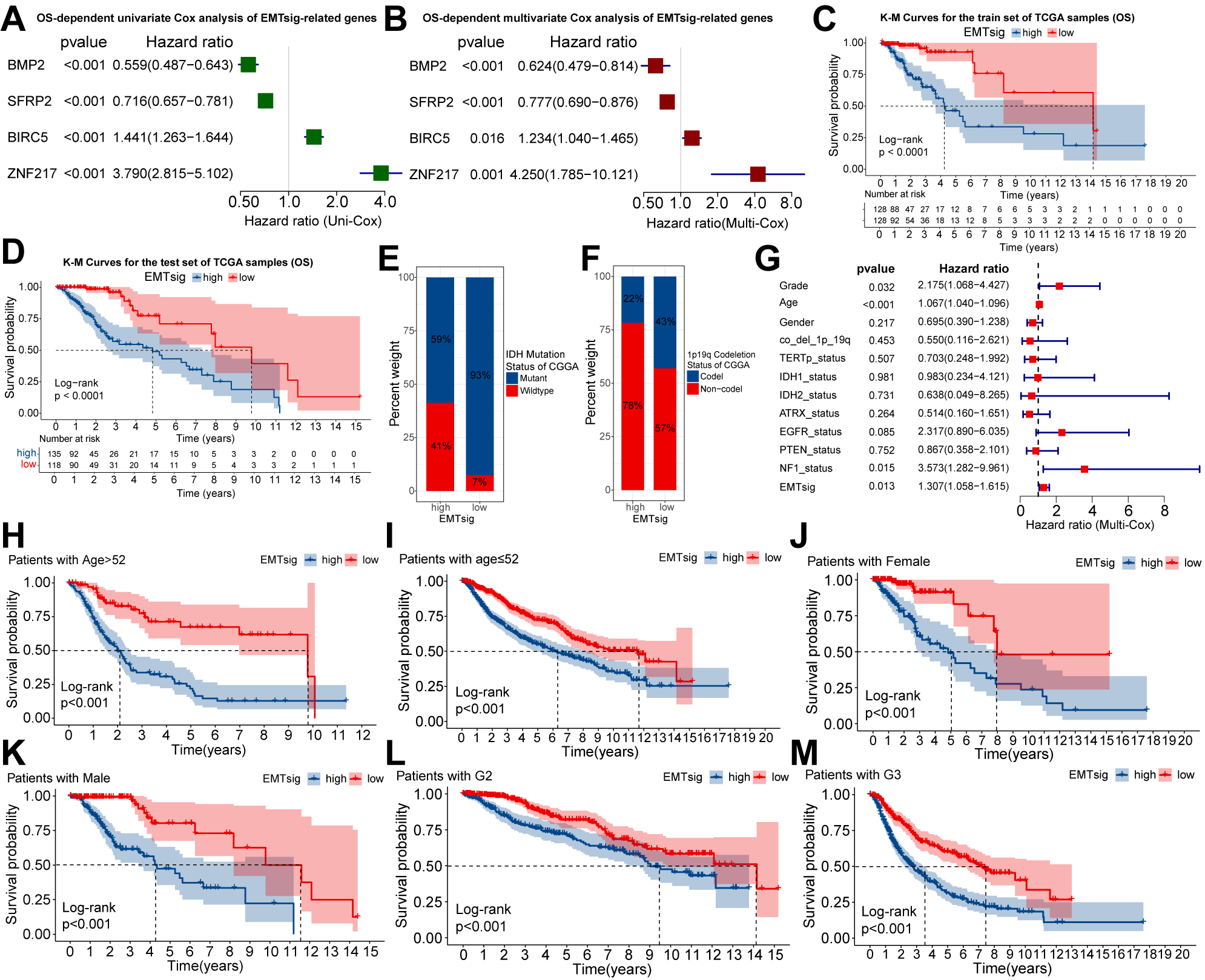


**Supplementary Figure S8:** Differences in EMTsig between C1 and C2 subtypes **(A)**. Differences in the distribution of the C1 and C2 subtypes between the high and low EMTsig subgroups **(B)**. Correlations of EMTsig with ESTIMATE score **(C)**, mDNAsi **(D)**, and mRNAsi **(E)**. GSVA results of activated GO **(F)** and KEGG **(G)** pathways in the low EMTsig subgroup of TCGA samples. GSVA results of activated GO **(H)** and KEGG **(I)** pathways in the high EMTsig subgroup of normal samples.


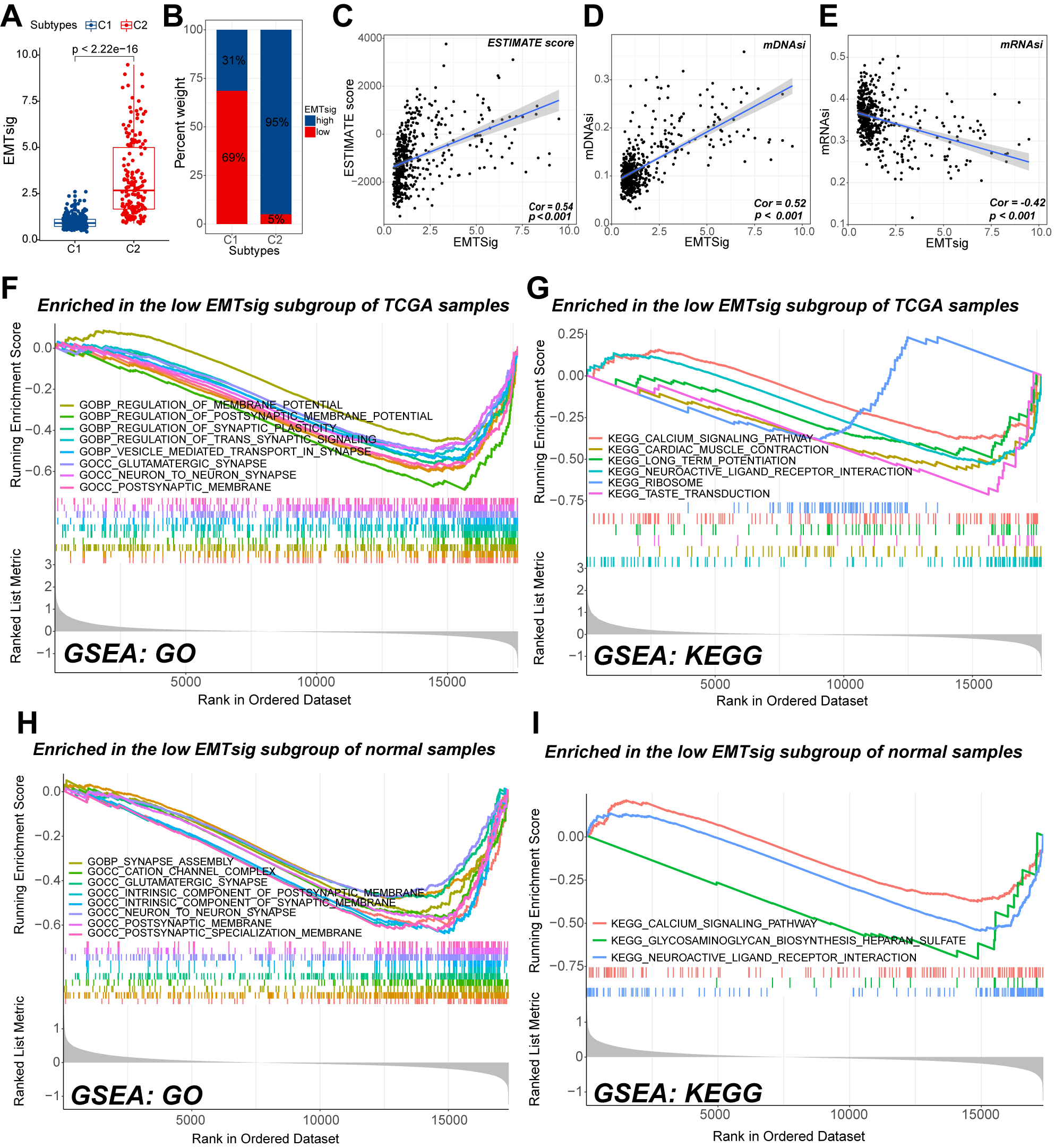


**Supplementary Figure S9:** Correlation of EMTsig-related genes with the sensitivity of chemotherapeutic agents **(A)** and molecular targeted drugs **(B)**. Correlation of EMTsig with tumor mutation burden (TMB) in the TCGA cohort **(C)**. Differences in the expression of immune checkpoints **(D)** and dysfunction score **(E)** between high and low EMTsig subgroups in the TCGA cohort. In the box plots, p<0.05 was indicated by "*”, p<0.01 was indicated by "**", p<0.001 was indicated by "***", and the statistical analysis was performed by the Mann-Whitney U test.


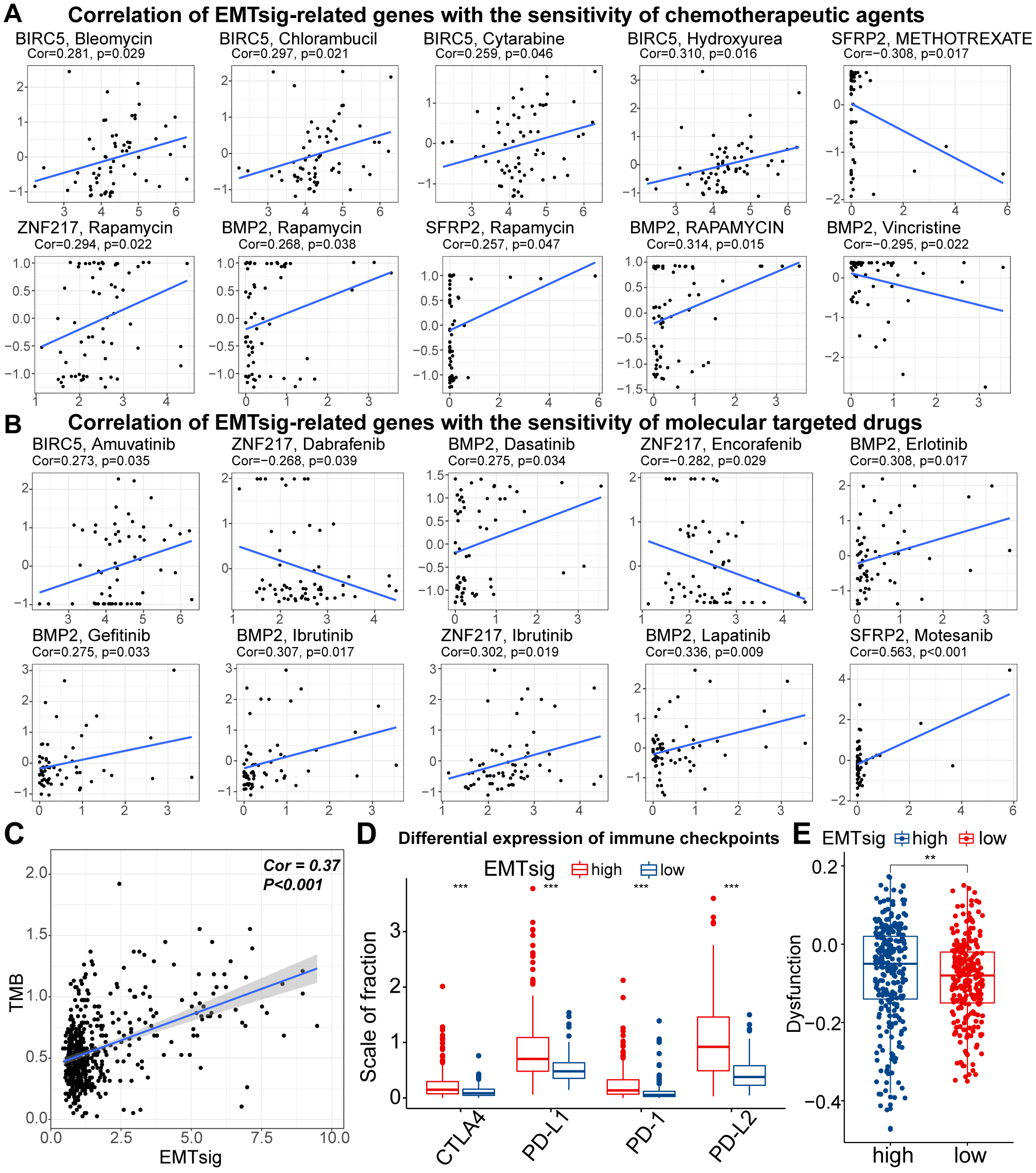

Supplement: Supplementary file 12 [file DataSheet1.DOCX]
